# Association of Clinical and Immunological Characteristics With Disease Severity and Outcomes in 211 Patients With COVID-19 in Wuhan, China

**DOI:** 10.3389/fcimb.2021.667487

**Published:** 2021-05-28

**Authors:** Man Wang, Yongzhen Fan, Yuqiong Chai, Wenlin Cheng, Kun Wang, Jianlei Cao, Xiaorong Hu

**Affiliations:** ^1^ Institute for Translational Medicine, The Affiliated Hospital of Qingdao University, College of Medicine, Qingdao University, Qingdao, China; ^2^ Department of Cardiology, Zhongnan Hospital of Wuhan University, Wuhan, China; ^3^ Institute of Myocardial Injury and Repair, Wuhan University, Wuhan, China

**Keywords:** COVID-19, clinical characteristics, immunological characteristics, disease severity, clinical outcome

## Abstract

**Background:**

Coronavirus disease 2019 (COVID-19) has posed a great threat to global public health. There remains an urgent need to address the clinical significance of laboratory finding changes in predicting disease progression in COVID-19 patients. We aimed to analyze the clinical and immunological features of severe and critically severe patients with COVID-19 in comparison with non-severe patients and identify risk factors for disease severity and clinical outcome in COVID-19 patients.

**Methods:**

The consecutive records of 211 patients with COVID-19 who were admitted to Zhongnan Hospital of Wuhan University from December 2019 to February 2020 were retrospectively reviewed.

**Results:**

Of the 211 patients with COVID-19 recruited, 111 patients were classified as non-severe, 59 as severe, and 41 as critically severe cases. The median age was obviously higher in severe and critically severe cases than in non-severe cases. Severe and critically severe patients showed more underlying comorbidities than non-severe patients. Fever was the predominant presenting symptom in COVID-19 patients, and the duration of fever was longer in critically severe patients. Moreover, patients with increased levels of serum aminotransferases and creatinine (CREA) were at a higher risk for severe and critical COVID-19 presentations. The serum levels of IL-6 in severe and critically severe patients were remarkably higher than in non-severe patients. Lymphopenia was more pronounced in severe and critically severe patients compared with non-severe patients. Lymphocyte subset analysis indicated that severe and critically severe patients had significantly decreased count of lymphocyte subpopulations, such as CD4+ T cells, CD8+ T cells and B cells. A multivariate logistic analysis indicated that older age, male sex, the length of hospital stay, body temperature before admission, comorbidities, higher white blood cell (WBC) counts, lower lymphocyte counts, and increased levels of IL-6 were significantly associated with predicting the progression to severe stage of COVID-19.

**Conclusion:**

Older age, male sex, underlying illness, sustained fever status, abnormal liver and renal functions, excessive expression of IL-6, lymphopenia, and selective loss of peripheral lymphocyte subsets were related to disease deterioration and clinical outcome in COVID-19 patients. This study would provide clinicians with valuable information for risk evaluation and effective interventions for COVID-19.

## Introduction

In December 2019, an outbreak of viral pneumonia, now known as coronavirus disease 2019 (COVID-19), was reported in Wuhan, Hubei Province, China (Huang et al., 2020). The etiological agent of COVID-19 was quickly identified as a novel coronavirus, severe acute respiratory syndrome coronavirus 2 (SARS-CoV-2), which is phylogenetically close to SARS-CoV (Zhou et al., 2020; Zhu et al., 2020). SARS-CoV-2 is regarded as the third highly pathogenic human coronavirus of the 21st century (Hu et al., 2020). However, the origin of COVID-19 has not been determined. SARS-CoV-2-infected patients, including asymptomatic carriers, are the primary source of infection (Rothe et al., 2020). The incubation period of COVID-19 ranges from 1 to 14 days and is commonly 3-7 days; therefore, it is difficult to identify infected patients in the early stage (Backer et al., 2020). SARS-CoV-2 is primarily transmitted by respiratory droplets and contact (Richard et al., 2020). Moreover, aerosol dispersal can take place in a relatively closed milieu (Zhang et al., 2020). Remarkably, SARS-CoV-2 enters host cells by the attachment of its spike protein to angiotensin-converting enzyme 2 (ACE2) located in the host cell membrane (Wan et al., 2020). Thus, SARS-CoV-2 can invade tissues with high expression levels of ACE2, such as the lungs, heart, liver, kidneys, intestines, and immune organs (Barton et al., 2020; Chen et al., 2020; Yang et al., 2020).

The outbreak of COVID-19 is ongoing and poses a great threat to public health. According to World Health Organization (WHO) data, there have been more than 102 million confirmed cases of COVID-19 worldwide as of 1 February 2021 with over 2.2 million related deaths, representing a fatality rate of approximately 2.2% (https://www.who.int/). Due to the high mortality rate and immense economic damage caused by the COVID-19 pandemic, great efforts are being made to develop effective vaccines/drugs to prevent and control the transmission of SARS-CoV-2. Thus, it is of paramount importance to clearly characterize the clinical, laboratory, and radiologic manifestations of this disease in large cohorts of patients. Previous studies have shown that COVID-19 patients can experience a range of clinical manifestations, from asymptomatic/mild symptoms to severe illness. Common symptoms include fever, dry cough, headache, shortness of breath, muscle soreness, fatigue, loss of taste and/or smell (Guan et al., 2020; Lovato et al., 2020). Severe interstitial pneumonia, acute respiratory distress syndrome (ARDS), multiple-organ dysfunction and death can occur in severe or critical cases (Geleris et al., 2020; Richardson et al., 2020; Zhou et al., 2020). The universal laboratory abnormalities in COVID-19 patients include increased inflammatory markers, lymphopenia, thrombocytopenia, and aberrant liver and renal functions (Goyal et al., 2020; Zhu et al., 2020).

Previously, the clinical and immunological characteristics of 21 patients (11 severe and 10 moderate cases) with COVID-19 were analyzed (Chen et al., 2020). Severe patients had decreased numbers of T lymphocyte subpopulations compared to moderate cases. The production of IFN-γ by CD4+ T cells was also suppressed in severe cases. These immunological characteristics might be associated with disease severity in COVID-19 patients. Wang et al. (2020) compared laboratory and immune indexes among 30 mild, 20 severe and 15 extremely severe patients with COVID-19. They found that the proportions of IFN-γ-producing T cells were higher in severe and extremely severe patients than in mild patients. Moreover, the serum levels of IL-2R, IL-6 and IL-10 were elevated in extremely severe patients. The function of T cell subsets might be related to disease progression in COVID-19 patients. Frequent fever, dry cough, and increased levels of inflammatory cytokines were shown to be the main clinical features in 69 patients with COVID-19 (Wang et al., 2020). In particular, older age and underlying comorbidities were associated with an increased risk of death in these patients. Zhang et al. (2020) identified multiple factors, including higher temperature, blood leukocyte and neutrophil counts, and aminotransferase and creatinine (CREA) activity, associated with the disease severity of COVID-19 through the analysis of clinical characteristics in 95 cases. The comparison of clinical characteristics in 43 non-severe and 26 severe patients showed that increased levels of IL-6 and IL-8 were correlated with disease deterioration (Li et al., 2020). In addition, lymphopenia and depletion of T lymphocytes were also observed in severe cases. The clinical data from 31 severe and 23 critical patients demonstrated that higher lymphocyte counts and increased levels of IL-2R and IL-6 were linked with predicting the progression to severe stage of COVID-19 (Li et al., 2020). In another study, a total of 123 patients were divided into mild (102) and severe (21) groups (Wan et al., 2020). There were significant differences in T lymphocyte subsets, IL-6 and IL-10 between the two groups. Likewise, a sustained decrease in lymphocyte subsets and proinflammatory cytokine storms were shown to significantly correlate with disease progression and poor prognosis in 67 patients with COVID-19 (Liu et al., 2021). In 35 patients with COVID-19, the decreased number of CD4+ T lymphocytes and the increased levels of TNF-α and IL-6 were related to the severity of COVID-19 disease (Sun et al., 2020). However, these studies only included a small number of patients. Thus, some results should be validated in future studies.

There have been some large-cohort studies that describe the clinical characteristics of COVID-19 patients. For example, Liao et al. (2020) analyzed the clinical features of 163 mild and 29 severe patients with COVID-19. They revealed that liver injury, elevated levels of inflammatory cytokines (IL-6, IL-10, and IL-17A) and a decline in the numbers of T lymphocyte subsets were commonly detected in the severe group. In a large multicentre, retrospective, cohort study involving 232 cancer patients and 519 patients without cancer, increased TNF-α and IL-6, as well as decreased lymphocytes and CD4+ T cells, were found to be risk factors for disease aggravation in patients with cancer and COVID-19 (Tian et al., 2020). A total of 1190 inpatients were enrolled in a retrospective, single-center cohort study (Liu et al., 2020b). Lymphocytopenia on admission and diabetes were identified to be potential predictors relevant to in-hospital deterioration and death. Although a number of published papers have indicated the clinical characteristics and imaging findings of COVID-19 patients, risk factors contributing to deterioration and poor outcome in severe and critically severe patients with COVID-19 have not been well described. The clinical significance of laboratory finding changes in predicting disease severity and clinical outcomes in COVID-19 patients warrants comprehensive investigation. In this study, we conducted a systematic analysis of the clinical and laboratory manifestations in 211 consecutive patients with COVID-19, and compared the variations among non-severe, severe and critically severe patients, uncovering the relationship between age, sex, body temperature, underlying comorbidities, serum biochemical parameters, lymphocyte subset profiles, inflammatory markers, and disease progression. The characterization of the indicative factors that may represent signs of disease severity in COVID-19 patients could be helpful for improving the management of this disease.

## Materials and Methods

### Patient Cohorts

Before March 13 2020, Zhongnan Hospital of Wuhan University received COVID-19 patients from isolation points, communities, and other hospitals. After this date, all hospitalized patients were transferred to Leishenshan Hospital for further medical treatment, adhering to the unified deployment strategy. This study covered 211 patients with laboratory confirmed COVID-19 disease from Zhongnan Hospital of Wuhan University in Wuhan, Hubei Province, China between 21 December 2019 and 14 February 2020. All patients were local residents of Wuhan. SARS-CoV-2 nucleic acids detection results were positive for all patients. Based on patients’ exposure history, clinical symptoms, laboratory examinations, and chest computed tomography (CT) scans, all patients received a clinical diagnosis of COVID-19 according to the WHO interim guidance. Moreover, some patients had a history of exposure to the Huanan Seafood Wholesale Market, or else had been in contact with people who had been diagnosed with COVID-19. The dates of disease onset and hospital admission were recorded. The onset date was defined as the day when any symptoms were noticed by the patients. Based on disease severity, the patients were divided into non-severe (total 111, male 38 and female 73), severe (total 41, male 23 and female 18) and critically severe (total 59, male 40 and female 19) groups according to the Diagnosis and Treatment of Novel Coronavirus Patients (the Fifth Pilot Ed.). There was no statistical significance in sex distribution among the three groups (*p*=0.276). The average ages of the non-severe, severe and critically severe groups were 47 ± 15.6, 60 ± 16.1, and 65 ± 14.8 years, respectively. Clinical characteristics, demographic information, laboratory examinations and chest CT scans of the patients were reviewed using electronic medical records. Laboratory examinations included routine blood tests, cytokine measurement, lymphocyte absolute values and lymphocyte subset analysis. The laboratory data for some patients were missing due to the absence of types of tests or delayed results. Patients in the non-severe, severe and critically severe groups were given corresponding treatment measures according to their clinical situation following admission, including antivirals, glucocorticoids, antibiotics, intravenous immunoglobulin, mechanical ventilation (**Supplementary Table 1**). The final date of follow-up was 13 March 2020.

### Clinical Samples

Peripheral venous blood samples from COVID-19 patients were obtained at admission, placed into separation gel vacuum procoagulant collective tubes and EDTA-anticoagulated vacutainer tubes, and then centrifuged at 400×g for 5 min at 4°C. Plasma samples were collected and stored at -80°C until use. EDTA-anticoagulated blood samples were used for routine blood tests and lymphocyte subset analysis. The biochemical indicators were examined using an automatic serum biochemical analyzer (ADVIA 2400, Siemens, Munich, Germany).

### Cytokine Measurement

The serum levels of six different cytokines (IFN-γ, TNF-α, IL-2, IL-4, IL-6 and IL-10) in COVID-19 patients were detected using a BD FACSCalibur flow cytometer (Becton, Dickinson and Company, New Jersey, USA) and commercially available cytokine kits (Node Company, Jiangxi, China) according to the manufacturers’ protocols. In brief, 25 µL of serum sample was mixed with capture antibody-coupled beads, and the mixture was then added to 25 µL fluorescently labeled detection reagent. The samples were incubated at room temperature in the dark for 2.5 h. The beads were subsequently washed and re-suspended in 100 µL sheath fluid and analyzed using flow cytometry. A recombinant protein standard for each cytokine was included to serve as an internal control.

### Lymphocyte Subset Analysis

Samples of EDTA-anticoagulated peripheral blood were collected from patients with COVID-19 on admission. The counts of CD3+ T cells, CD4+ T cells, CD8+ T cells, B cells and NK cells were analyzed on a BD FACSCalibur flow cytometer.

### Statistical Analysis

Categorical variables were shown as frequencies and percentages, and continuous variables were described with the mean ± standard deviation (SD) or median (interquartile range, IQR) as appropriate. All analyses were carried out using SPSS software (Version 24.0; IBM Corporation, Armonk, New York). The non-normally distributed data were analyzed using the Kruskal-Wallis test. The enumeration data were analyzed using the χ^2^ test or Fisher’s exact test. The Pearson correlation test was performed to analyze correlations between lymphocyte subset counts and cytokine levels. Similarly, correlations between peripheral lymphocyte subpopulations and disease progression were constructed using Pearson correlation analysis. Receiver operating characteristic (ROC) curves were established to define the optimal cut-off values. The area under the curve (AUC) was used to compare the predictive ability of peripheral lymphocyte subpopulations. The demographics and laboratory indexes were assessed by multivariable logistic regression analyses to explore the independent predictors and risk factors for severe (*vs*. non-severe) and critical (*vs*. non-severe) diseases in COVID-19 patients. Age, sex, the duration from symptom onset to hospital admission, the length of hospital stay, body temperature, comorbidity, complete blood count, liver and renal function indicators, inflammatory cytokines, lymphocyte subsets, and T cell subsets were included in the multivariable logistic regression model. A two-sided *p* value less than 0.05 was considered to be statistically significant.

## Results

### Demographic and Clinical Characteristics of Patients With COVID-19

From 21 December 2019 to 14 February 2020, 211 patients with confirmed SARS-CoV-2 infection were hospitalized with a median follow-up of 13 days (range, 0-50 days). The endpoint of follow-up was 13 March 2020. The demographic and clinical characteristics of the COVID-19 patients were shown in **Table 1**. Of the 211 patients hospitalized with COVID-19, 41 (19.4%) had severe disease, and 59 (28.0%) had critical disease. The median age was 54 years (IQR, 41-67; range, 7-96 years). The age distribution showed that 121 (57.3%) patients were aged <60 years, and the other patients were aged ≥60 years (**Supplementary Figure 1**). Among them, 64 (30.3%) were aged 30-49 years, and 87 (41.2%) were aged 50-69 years. Moreover, 43 (20.4%) patients were aged 70 years or over. Compared with non-severe patients, severe patients with COVID-19 were significantly older [median age, 60 (IQR, 51-69) *vs*. 47 (IQR, 34-58) years, *p*<0.001; **Supplementary Figure 1**]. The median age was obviously higher in critically severe cases than in non-severe cases [median age, 65 (IQR, 55-76) *vs*. 47 (IQR, 34-58) years, *p*<0.001]. Additionally, 101 patients (47.9%) were male, and 110 patients (52.1%) were female. The proportions of men in the severe (56.1%) and critically severe (67.8%) cases were higher than those in the mild cases (34.2% men). Male patients had more underlying comorbidities than female patients [43 (42.6%) *vs*. 31 (28.2%), *p*<0.001; **Supplementary Table 2**]. Thus, male COVID-19 patients were more likely to suffer from severe diseases. It has been proposed that sex-related differences in physiology, the immune system and sex hormone milieu may be contributing factors to disease severity in males (Samuel et al., 2020; Takahashi et al., 2020; Rastrelli et al., 2021). The median time from onset of symptoms to admission was 7.1 days (IQR, 4.0-10.0 days). The duration of symptoms before admission was also longer in severe and critically severe patients (7.0 *vs*. 6.3 days, *p*=0.536; 8.9 *vs*. 6.3 days, *p*<0.01). Of the 211 patients with COVID-19, less than half [74 (35.1%)] had at least one underlying disease, including hypertension (53 [25.1%]), diabetes [28 (13.3%)], cardiovascular and cerebrovascular diseases [25 (11.8%)] and respiratory system diseases [15 (7.1%)]. A higher percentage of severe [22 (53.7%)] and critically severe [36 (61.0%)] patients had these concomitant diseases than the non-severe group [16 (14.4%)]. Severe patients were more likely to have accompanying hypertension and diabetes than non-severe patients [16 (39.0%) *vs*. 11 [9.9%], *p*<0.001; and 10 [24.4%] *vs*. 4 [3.6%], *p*=0.001; respectively]. Critically severe patients showed more underlying comorbidities than non-severe patients, such as hypertension [26 (44.1%) *vs*. 11 (9.9%), *p*<0.001], diabetes [14 (23.7%) *vs*. 4 (3.6%), *p*<0.001] and cardiovascular and cerebrovascular diseases [18 (30.5%) *vs*. 2 (1.8%), *p*<0.001].

**Table 1:** Main clinical characteristics of 211 patients with COVID-19.

	All (n=211)	Disease severity					*p* value
		Non-severe (n=111)		Severe (n=41)		Critically Severe (n=59)	
**Characteristics**							
Age, years	54 (41-67)	47 (34-58)		60 (51-69)		65 (55-76)	<0.001
30-49	64 (30.3%)	48 (43.2%)		7 (17.1%)		9 (15.3%)	0.259
50-69	87 (41.2%)	41 (36.9%)		22 (53.7%)		24 (40.7%)	–
≥70	43 (20.4%)	8 (7.2%)		10 (24.4%)		25 (42.4%)	–
**Sex**							
Male	101 (47.9%)	38 (34.2%)		23 (56.1%)		40 (67.8%)	0.276
Female	110 (52.1%)	73 (65.8%)		18 (43.9%)		19 (32.2%)	–
**Epidemiological history**							
Family associated with COVID-19	11 (5.2%)	7 (6.3%)		3 (7.3%)		1 (1.7%)	0.349
**Timeline after onset of illness, days**							
Median time from illness onset to admission	7.1 (4.0-10.0)	6.3 (3-9)		7.0 (3-9)		8.9 (5-11)	0.012
Median follow-up time from admission	13.3 (8-19)	11.8 (8-15)		14.4 (10-22)		15.2 (10-24)	0.121
**Temperature before admission, °C**	38.0 ± 1.0	37.8 ± 1.0		38.3 ± 0.9		38.2 ± 1.0	0.001
Highest temperature	40 (37.3-38.9)	39.5 (36.6-38.5)		40 (37.8-39)		39.9 (37.8-39)	0.368
<37.3	49 (23.2%)	32 (28.8%)		5 (12.2%)		12 (20.3%)	0.204
37.3-38	62 (29.4%)	38 (34.2%)		11 (26.8%)		13 (22.0%)	–
38.1-39	76 (36.0%)	34 (30.6%)		18 (43.9%)		24 (40.7%)	–
>39	24 (11.4%)	7 (6.3%)		7 (17.1%)		10 (16.9%)	–
**Temperature at day 15 after admission, °C**	36.8 ± 0.9	36.6 ± 0.4		36.6 ± 0.4		37.2 ± 1.2	0.006
Highest temperature	41.7 (36.4-37.1)	37.3 (36.3-36.7)		37.4 (36.3-36.8)		41.7 (36.5-37.3)	0.368
<37.3	79 (84.0%)	30 (93.8%)		21 (91.3%)		28 (71.8%)	0.19
37.3-38	9 (9.6%)	2 (6.3%)		2 (8.7%)		5 (12.8%)	–
38.1-39	4 (4.3%)	0 (0.0%)		0 (0.0%)		4 (10.3%)	–
>39	2 (2.1%)	0 (0.0%)		0 (0.0%)		2 (5.1%)	–
**Comorbidity**							
Hypertension	53 (25.1%)	11 (9.9%)		16 (39.0%)		26 (44.1%)	<0.001
Diabetes	28 (13.3%)	4 (3.6%)		10 (24.4%)		14 (23.7%)	<0.001
Cardiovascular and cerebrovascular diseases	25 (11.8%)	2 (1.8%)		5 (12.2%)		18 (30.5%)	<0.001
Respiratory system diseases	15 (7.1%)	4 (3.6%)		3 (7.3%)		8 (13.6%)	0.055
**Clinical outcome**							
Remained in hospital	14 (6.6%)	1 (0.9%)		5 (12.2%)		8 (13.6%)	0.002
Transferred	3 (1.4%)	3 (2.7%)		0 (0.0%)		0 (0.0%)	0.256
Discharged	165 (78.2%)	107 (96.4%)		36 (87.8%)		22 (37.3%)	<0.001
Died	29 (13.7%)	0 (0.0%)		0 (0.0%)		29 (49.2%)	<0.001

Data are expressed as median (IQR), n (%), or mean ± SD. P values comparing non-severe, severe and critically severe groups are from χ^2^ test, Fisher’s exact test, or Kruskal-Wallis test. P<0.05 was considered as statistically significant.

**Figure 1:**
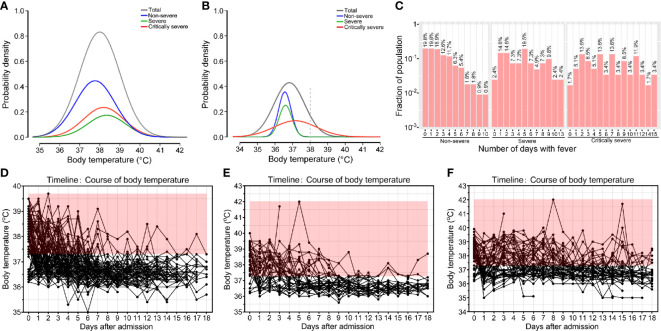
Body temperatures of patients with COVID-19. Probability density of temperatures of non-severe, severe, critically severe patients with COVID-19 on admission **(A)** and on day 15 after admission **(B)**. **(C)** Histogram of the number of COVID-19 patients that developed fever. Temperature curves of non-severe **(D)**, severe **(E)** and critically severe patients **(F)** during hospitalization. Body temperature ≥ 37.3°C is shown in red.

The most common clinical feature of these patients at the onset of illness was fever [162 (76.8%)]. Among the patients, 76 (36.0%) had a temperature varying from 38.1 to 39.0°C, and 24 (11.4%) had a temperature that exceeded 39.0°C before hospital admission. Seventy-nine (71.2%) non-severe patients reported fever, 36 (87.8%) severe patients and 47 (79.7%) critically severe patients developed fever. As shown in **Figure 1A**, the body temperatures of severe and critically severe patients were significantly higher than those of non-severe patients before hospital admission (38.3 *vs*. 37.8°C, *p*<0.01; 38.2 *vs*. 37.8°C, *p*<0.01). The distribution density curves of temperatures substantially overlapped among these groups. Following hospital admission, critically severe patients had a higher percentage of fever cases [27 (45.8%)] than non-severe [9 (8.1%)] and severe [8 (19.5%)] patients. The cases of fever fell to 15 (16.0%) at 15 days after admission. In critically severe cases, 11 (28.2%) patients had a temperature over 37.3°C, with four (10.3%) reporting temperatures within 38.1-39.0°C and two (5.1%) exceeding 39°C. Only a minority of patients presented with fever in the non-severe and severe groups (2/32; 2/23). At 15 days after admission, the median body temperature of critically severe patients was significantly greater than that of non-severe patients (37.2 *vs*. 36.6°C, *p*<0.01; **Figure 1B**). The body temperature of severe patients (36.6°C) was comparable to that of non-severe patients (36.6°C).

Moreover, 11.4% (24/211) of COVID-19 patients never had fever, and 73.0% (154/211) suffered from fever for no longer than five days. A total of 89.2% (99/111) of non-severe patients had fever lasting no more than five days (**Figure 1C**). Among severe and critically severe patients, 65.9% (27/41) and 47.5% (28/59) presented fever for no more than five days, respectively. Notably, 14 out of 59 (23.7%) critically severe patients presented with a continued fever for at least ten days. Most non-severe (110/111, 99.1%) and severe (39/41, 95.1%) patients had no fever at 13 days after admission (**Figures 1D, E**). However, 27.9% (12/43) of critically severe patients still had fever 13 days after admission (**Figure 1F**). Overall, compared with non-severe and severe patients, critically severe patients had a longer duration of fever.

**Figure 2:**
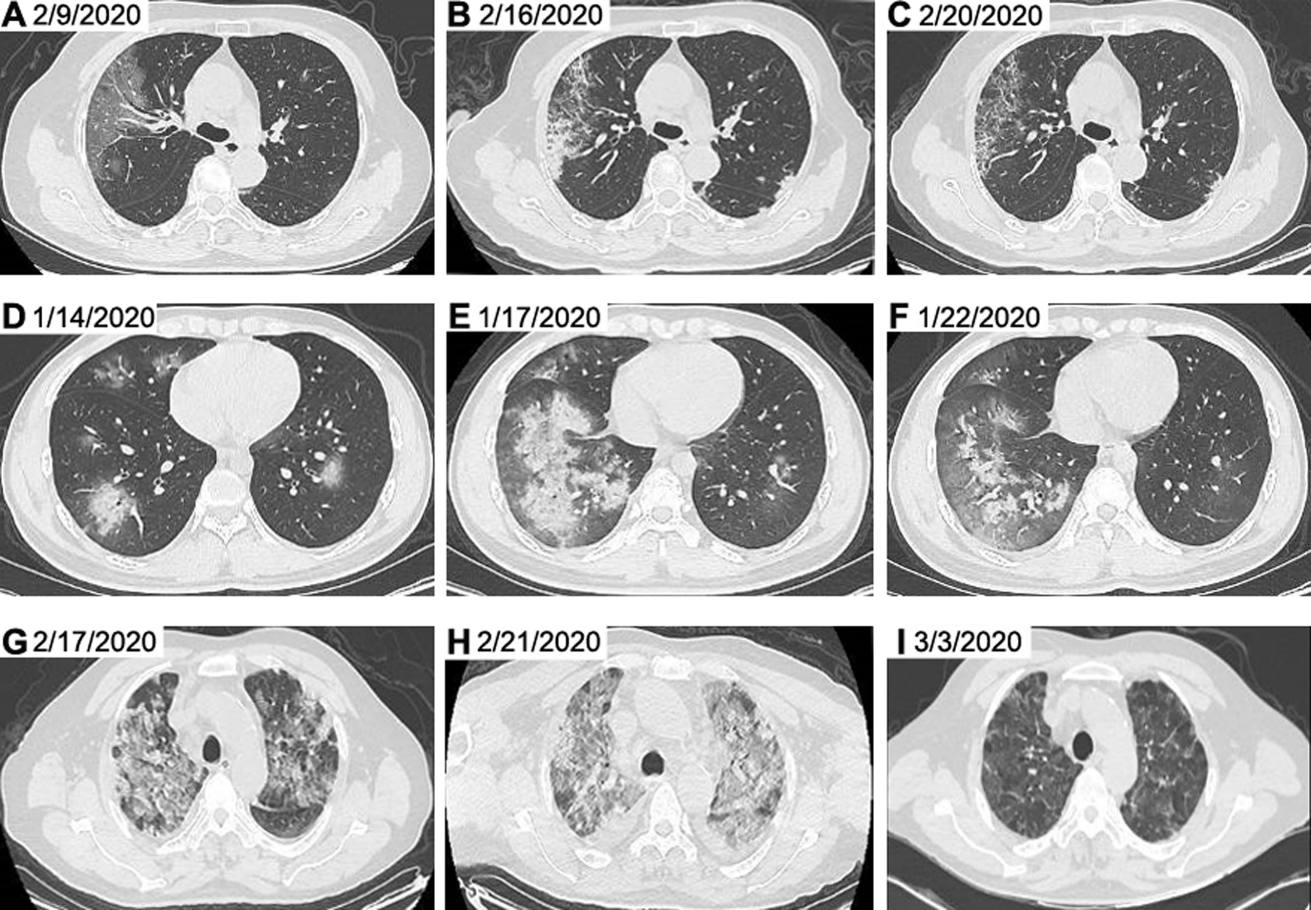
Representative chest CT images of patients with COVID-19. Chest CT images from a 65-year-old woman with non-severe disease on day 9 after symptom onset **(A)**, on day 16 after symptom onset **(B)** and on day 20 after symptom onset **(C)** were shown. Chest CT images from a 23-year-old man with severe disease on day 7 after symptom onset **(D)**, on day 10 after symptom onset **(E)** and on day 15 after symptom onset **(F)** were presented. Chest CT images from a 62-year-old man with critically severe disease on day 13 after symptom onset **(G)**, on day 17 after symptom onset **(H)** and on day 28 after symptom onset **(I)** were shown. The chest CT images indicated multi-focal ground glass opacity and parenchyma consolidation, mainly involving subpleural regions of both lungs in patients with COVID-19. CT, computed tomography.

At the time of admission, patients with COVID-19 had abnormal chest CT findings. A typical evolution of chest CT images of non-severe, severe and critically severe patients was shown in **Figure 2**. The non-severe patient showed ground-glass opacity along the outer bands of both lungs on day 9 after symptom onset (**Figure 2A**). Severe days later, the ground glass opacity began to solidify (**Figures 2B, C**). The opacity area of the ground glass was larger, and the consolidation degree was more serious in severe (**Figures 2D–F**) and critically severe patients (**Figures 2G, H**). Consolidation was gradually absorbed in critically severe patients as the disease progressed (**Figure 2I**). By 13 March 2020, 165 of 211 (78.2%) patients had been discharged, and 29 patients had died, with a mortality rate of 13.7% in this cohort. There were no deaths in non-severe and severe patients, while the mortality rate in critically severe patients was 49.2%. The median age of non-survivors was significantly higher than that of survivors (*p*<0.01; **Supplementry Figure 1**). The survival curve of the 59 critically severe patients showed that most deaths (23, 39.0%) occurred within 28 days after the onset of illness (**Supplementary Figure 2**).

### Laboratory Findings

**Figure 3:**
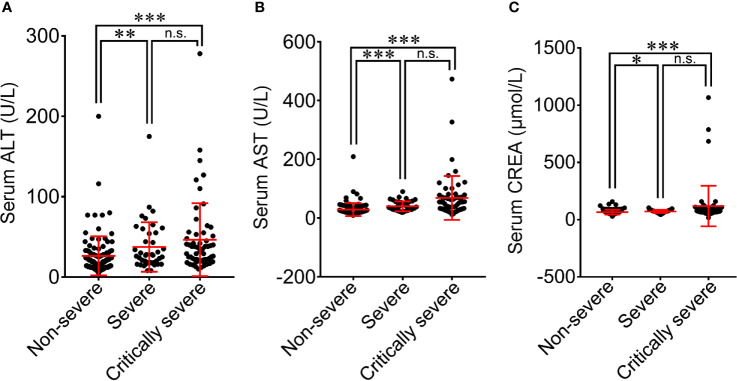
Comparison of blood biochemical parameters among non-severe, severe and critically severe patients with COVID-19. The levels of ALT **(A)**, AST **(B)** and CREA **(C)** were compared among non-severe, severe and critically severe patients. ALT, alanine aminotransferase; AST, aspartate aminotransferase; CREA, creatinine. *p<0.05; **p<0.01; ***p<0.001; n.s., not significant.

All patients with COVID-19 underwent laboratory examinations on admission (**Table 2**). The serum levels of alanine aminotransferase (ALT) and aspartate aminotransferase (AST) were in the normal range for most patients (169/209; 125/209), with 39 (18.7%) and 80 (38.3%) patients having higher ALT and AST levels than normal (**Supplementary Figure 3**). The serum level of CREA was within the normal range for the majority of COVID-19 patients (151/209, 72.2%). The increase in ALT, AST and CREA levels was more obvious in severe patients than in non-severe patients (37.4 *vs*. 26.4 U/L, *p*<0.01; 39.9 *vs*. 28.9 U/L, *p*<0.001; 71.9 *vs*. 65.7 μmol/L, *p*<0.05; **Figures 3A–C**). The critically severe group had significantly higher serum levels of ALT, AST and CREA than the non-severe group (46.4 *vs*. 26.4 U/L, *p*<0.001; 68.3 *vs*. 28.9 U/L, *p*<0.001; 119.7 *vs*. 65.7 μmol/L, *p*<0.001). There were no significant differences in the serum levels of ALT, AST or CREA between the severe and critically severe groups.

**Table 2:** Laboratory characteristics of 211 patients with COVID-19 on admission to hospital.

Laboratory findings	Normal range	All (n=211)	Non-severe (n=111)	Severe (n=41)	Critically severe (n=59)	*p* value
**Blood routine**						
White blood cell count, ×10^9^/L	3.5-9.5	5.9 (3.3-6.8)	4.6 (3.1-5.7)	5.6 (3.5-7.1)	8.5 (3.6-12.2)	<0.001
<3.5	–	62 (29.4%)	38 (34.2%)	10 (24.4%)	14 (23.7%)	0.708
3.5-9.5	–	122 (57.8%)	68 (61.3%)	26 (63.4%)	28 (47.5%)	–
>9.5	–	27 (12.8%)	5 (4.5%)	5 (12.2%)	17 (28.8%)	–
Lymphocyte count, ×10^9^/L	1.1-3.2	0.9 (0.6-1.2)	1.1 (0.7-1.3)	0.8 (0.5-1.0)	0.7 (0.4-0.9)	<0.001
<1.1	–	151 (71.6%)	65 (58.6%)	35 (85.4%)	51 (86.4%)	<0.001
1.1-3.2	–	59 (28.0%)	45 (40.5%)	6 (14.6%)	8 (13.6%)	–
>3.2	–	1 (0.5%)	1 (0.9%)	0	0	–
**Liver and renal function tests**						
Alanine aminotransferase, U/L	7-45	34.1 (16-38)	26.4 (14-28)	37.4 (17-54)	46.4 (21-51)	<0.001
≤40	–	161 (77.0%)	95 (86.4%)	28 (68.3%)	38 (65.5%)	0.004
>40	–	48 (23.0%)	15 (13.6%)	13 (31.7%)	20 (34.5%)	–
Aspartate aminotransferase, U/L	13-35	42.0 (21-46)	28.9 (19-32)	39.9 (26-54)	68.3 (30-71)	<0.001
≤35	–	129 (61.7%)	87 (79.1%)	21 (51.2%)	21 (36.2%)	<0.001
>35	–	80 (38.3%)	23 (20.9%)	20 (48.8%)	37 (63.8%)	–
Creatinine, µmol/L	49-90	81.9 (55.1-83.6)	65.7 (52.1-74.1)	71.9 (60-81.7)	119.7 (65-97.1)	<0.001
**Inflammatory cytokines**						
IFN-γ, pg/mL	0.1-18	1.6 (0.1-2.0)	1.9 (0.1-0.9)	2.9 (0.3-7.6)	1.2 (0.1-1.9)	0.408
TNF-α, pg/mL	0.1-23	1.2 (0.1-0.5)	2.8 (0.1-0.3)	0.7 (0.1-1.8)	0.5 (0-0.7)	0.514
IL-2, pg/mL	0.1-4.1	1.1 (0.2-1.0)	1.7 (0.1-1.0)	1.5 (0.2-2.9)	0.6 (0.2-0.7)	0.296
IL-4, pg/mL	0.1-3.2	0.7 (0.1-0.6)	1.3 (0.1-0.6)	0.5 (0.3-0.8)	0.4 (0-0.4)	0.064
IL-6, pg/mL	0.1-2.9	72.0 (8.5-80.9)	21.8 (2.4-22.6)	33.3 (9.5-54.7)	140.6 (17.5-130.1)	<0.001
IL-10, pg/mL	0.1-5.0	8.8 (2.1-6.0)	14.8 (1.1-3.1)	3.1 (1.1-3.9)	6.8 (2.2-8.6)	0.023

Data are expressed as median (IQR) or n (%). P values comparing non-severe, severe and critically severe groups are from χ^2^ test, Fisher’s exact test, or Kruskal-Wallis test. P<0.05 was considered as statistically significant.

### Relationship Between ALT, AST or CREA and Underlying Diseases of Patients With COVID-19

**Figure 4:**
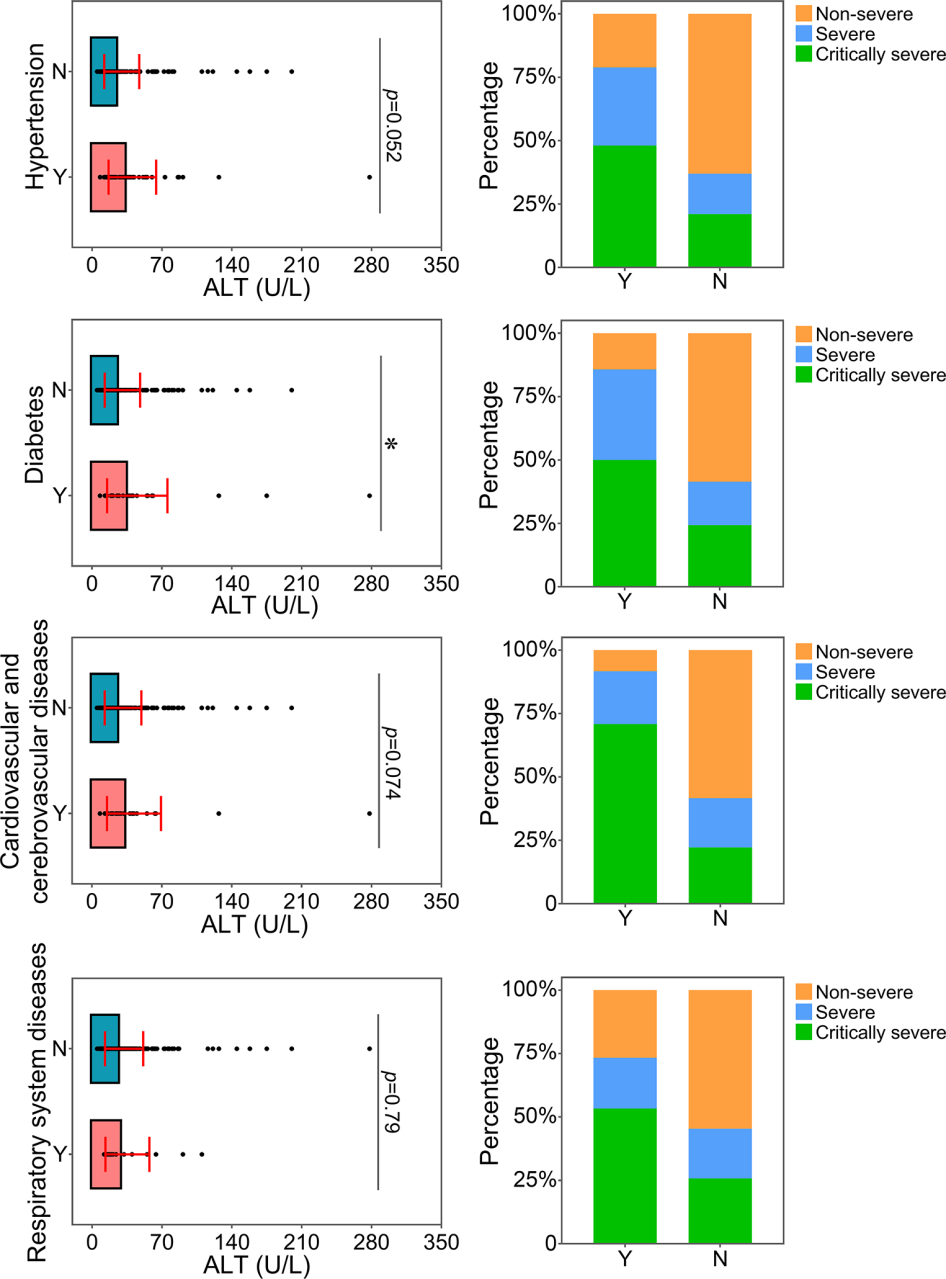
Relationship between ALT and underlying diseases. The ALT levels of patients with or without underlying diseases were compared. The percentage of COVID-19 classifications in each group was compared. N, patients without underlying disease; Y, patients with underlying disease. *p<0.05.

**Figure 5:**
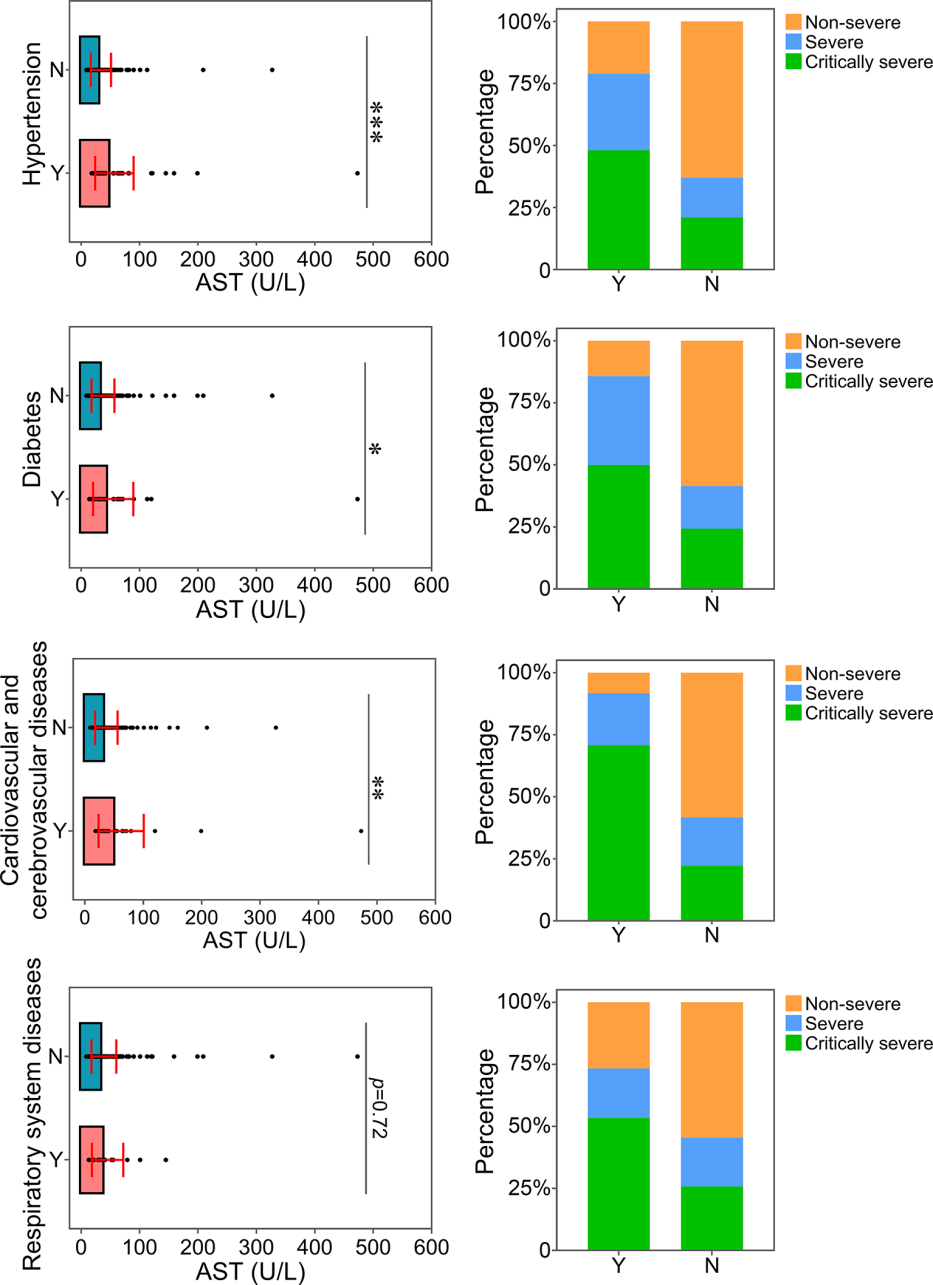
Relationship between AST and underlying diseases. The AST levels of patients with or without underlying diseases were compared. The percentage of COVID-19 classifications in each group was compared. N, patients without underlying disease; Y, patients with underlying disease. *p<0.05; **p<0.01; ***p<0.001.

**Figure 6:**
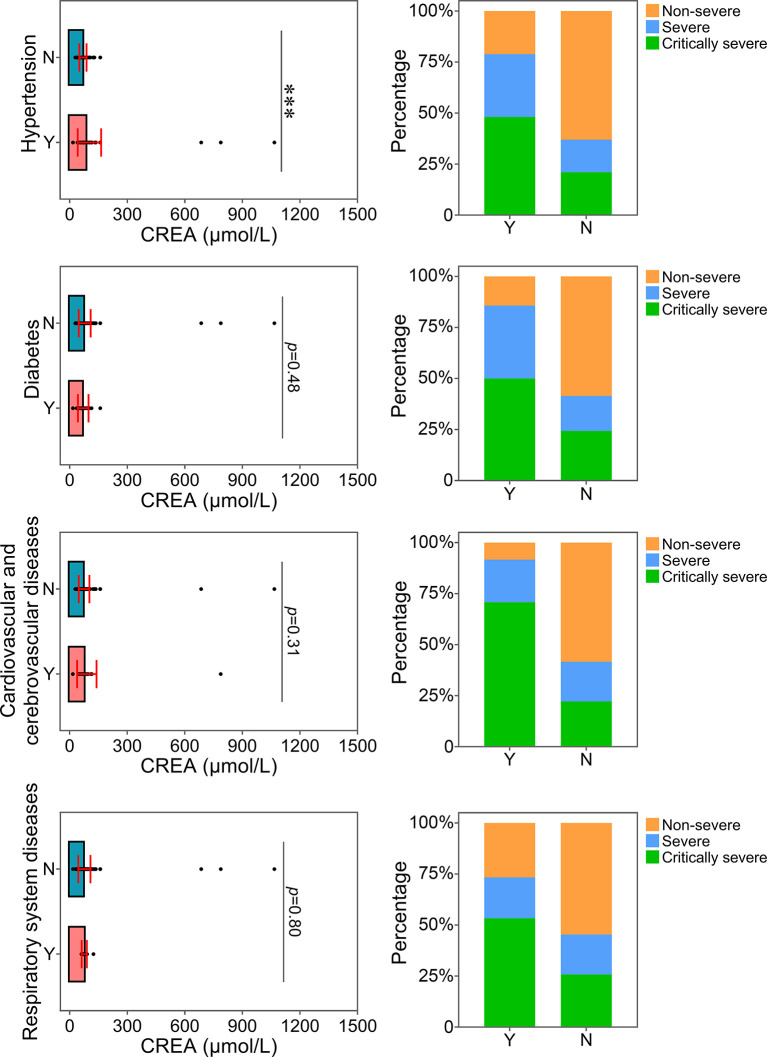
Relationship between CREA and underlying diseases. The CREA levels of patients with or without underlying diseases were compared. The percentage of COVID-19 classifications in each group was compared. N, patients without underlying disease; Y, patients with underlying disease. ***p<0.001.

The relationship between the levels of ALT, AST or CREA and underlying diseases was determined. Diabetes, one of the most common comorbidities, was tightly associated with disease progression in COVID-19 patients (Fang et al., 2020; Ma and Holt, 2020). Patients with diabetes were found to have significantly higher levels of ALT than patients without this disease (**Figure 4**). Patients with diabetes had a higher risk of disease progression. Moreover, the severity of COVID-19 was correlated with other comorbidities, including hypertension, cardiovascular and cerebrovascular diseases, and respiratory system diseases. Patients with these underlying diseases had higher ALT levels and were susceptible to disease progression. As shown in **Figure 5**, comorbidities including hypertension, diabetes, and cardiovascular and cerebrovascular diseases could increase the levels of AST and the deterioration of COVID-19 disease. The serum levels of CREA were markedly increased in patients with hypertension compared with those without this disease (**Figure 6**). A higher level of CREA was related to disease severity in patients with hypertension and COVID-19.

### Cytokine Profiles

**Figure 7:**
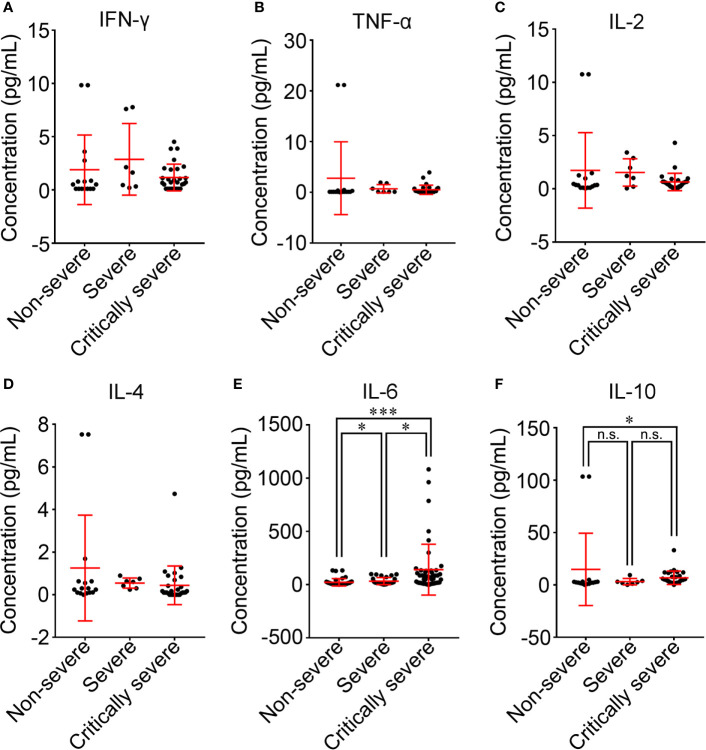
Comparison of cytokine levels among non-severe, severe and critically severe patients. The concentrations of IFN-γ **(A)**, TNF-α **(B)**, IL-2 **(C)**, IL-4 **(D)**, IL-6 **(E)** and IL-10 **(F)** in the serum of COVID-19 patients were compared among non-severe, severe and critically severe groups. The longer horizontal line represents the median value for each group. IFN-γ, interferon-γ; TNF-α, tumor necrosis factor-α; IL-2, interleukin-2; IL-4, interleukin-4; IL-6, interleukin-6; IL-10, interleukin-10. *p<0.05; ***p<0.001; n.s., not significant.

The cytokine profile of COVID-19 patients was analyzed at hospital admission. The mean values of IFN-γ in all groups were within the normal range and were not significantly different among the three groups (**Figure 7A**). The majority of COVID-19 patients had normal levels of TNF-α (40/52, 76.9%), IL-2 (47/52, 90.4%) and IL-4 (36/52, 69.2%) (**Supplementary Figure 4**). Compared with non-severe patients, TNF-α, IL-2 and IL-4 were decreased in severe and critically severe patients, and there was no significant difference among the three groups (*p*=0.514, *p*=0.296, *p*=0.064; **Figures 7B–D**). Ninety-nine of 115 (86.1%) COVID-19 patients had elevated levels of IL-6. The median concentration of IL-6 in the three groups exceeded the upper limit of the normal range (**Table 2**). As shown in **Figure 7E**, the increase in the serum level of IL-6 had a more obvious trend in the critically severe group than in the non-severe and severe groups (140.6 *vs*. 21.8 pg/mL, *p*<0.001; 140.6 *vs*. 33.3 pg/mL, *p*<0.05). The IL-10 level was within the reference range in 34 of 52 (65.4%) COVID-19 patients (**Supplementary Figure 4**). The median value of IL-10 in severe cases (3.1 pg/mL) was within the normal range, while the IL-10 level was above the upper limit of the normal range in non-severe and critically severe cases (14.8 and 6.8 pg/mL, respectively). Moreover, the serum level of IL-10 in the critically severe group was remarkably lower than that in the non-severe group (6.8 *vs*. 14.8 pg/mL, *p*<0.05; **Figure 7F**).

### Relationship Between IL-6 and Underlying Illnesses of Patients With COVID-19

**Figure 8:**
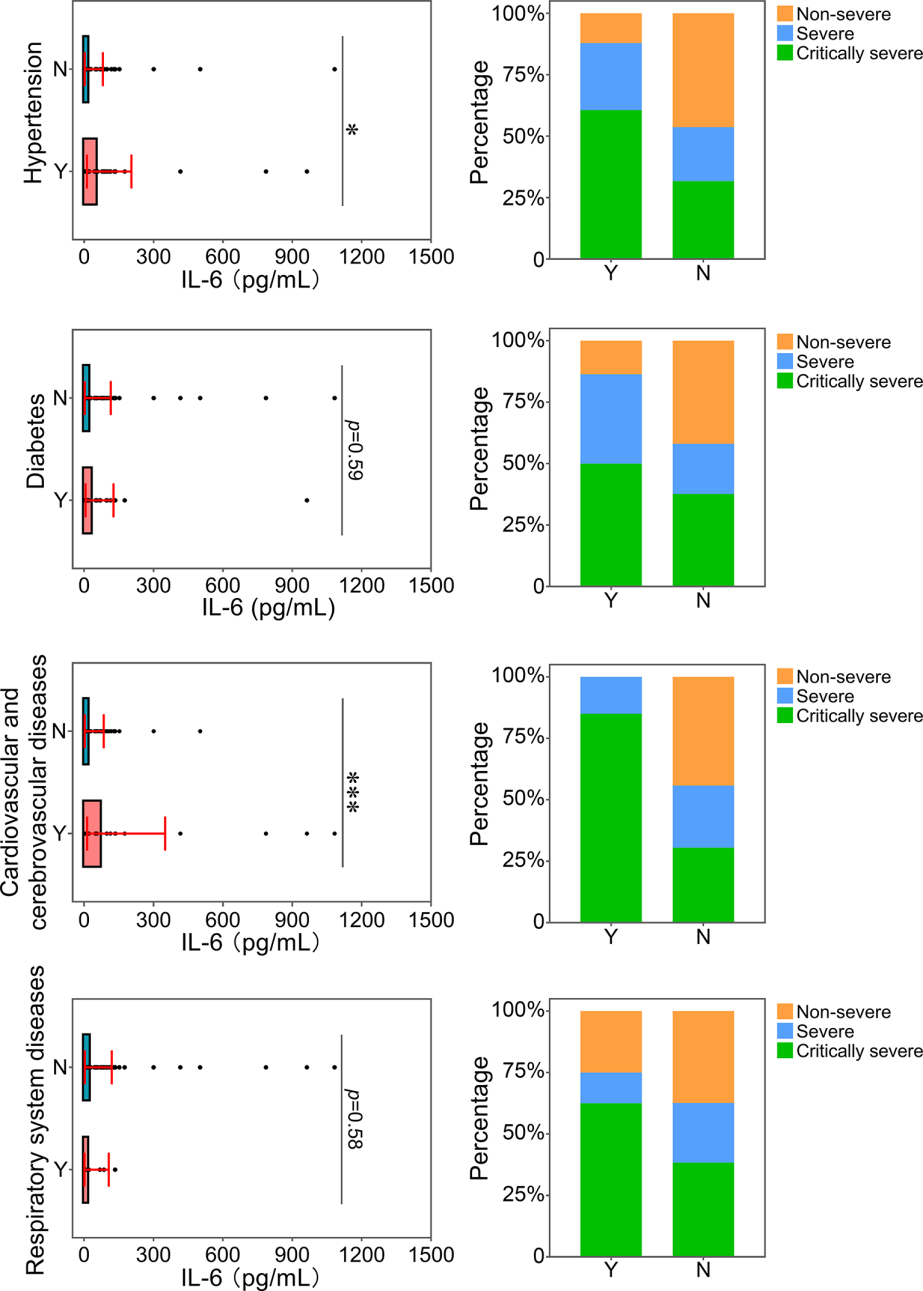
Relationship between IL-6 and underlying diseases. The IL-6 levels of patients with or without underlying diseases were compared. The percentage of COVID-19 classifications in each group was compared. N, patients without underlying disease; Y, patients with underlying disease. *p<0.05; ***p<0.001.

The correlation between the level of IL-6 and underlying diseases was further analyzed. Patients with underlying diseases were prone to develop severe and critical diseases (**Figure 8**). In particular, patients with hypertension and cardiovascular and cerebrovascular diseases had more serious inflammatory conditions, as indicated by higher levels of IL-6. Thus, these two kinds of comorbidities were related to an increase in the severity of inflammation in patients with COVID-19. In addition, the proportions of patients with hypertension and cardiovascular and cerebrovascular diseases in the severe and critically severe groups were higher than those in the non-severe group. Based on our comparison results, hypertension and cardiovascular and cerebrovascular diseases were closely associated with disease progression in COVID-19 patients, which could be linked with the increased level of IL-6. Reportedly, elevated IL-6 level is a risk factor for coronary heart disease (CHD) (Danesh et al., 2008). The proportion of patients with CHD and increased IL-6 was analyzed. The majority (20/25, 80%) of patients with cardiovascular and cerebrovascular diseases and COVID-19 showed elevated IL-6 levels. Of the 20 patients, 3 (15.0%) and 17 (85.0%) presented with severe and critical diseases, respectively. Only one of 17 (5.9%) critically severe patients developed CHD. Due to a lack of cases, the association between IL-6 and clinical outcome in patients with CHD and COVID-19 remains to be determined.

### Peripheral Lymphocyte Subpopulation Profiles

**Figure 9:**
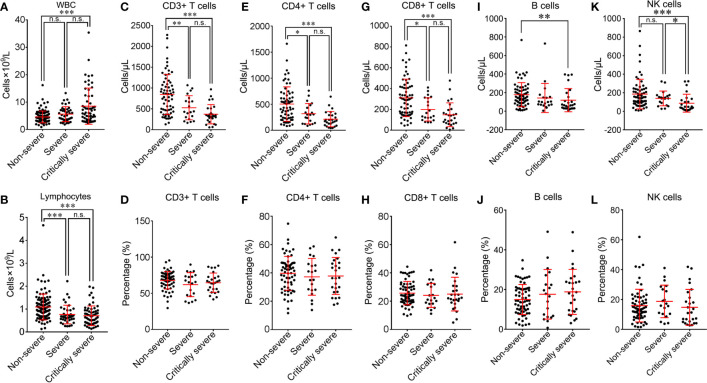
Comparison of peripheral lymphocyte subsets among non-severe, severe and critically severe patients. Absolute cell counts of WBC **(A)**, lymphocytes **(B)**, CD3+ T cells **(C)**, CD4+ T cells **(E)**, CD8+ T cells **(G)**, B cells **(I)** and NK cells **(K)** in non-severe, severe and critically severe patients were analyzed by flow cytometry. The percentages of CD3+ T cells **(D)**, CD4+ T cells **(F)**, CD8+ T cells **(H)**, B cells **(J)** and NK cells **(L)** were compared among non-severe, severe and critically severe patients. WBC, white blood cell; NK, natural killer. *p<0.05; **p<0.01; ***p<0.001; n.s., not significant.

There were many differences in white blood cell (WBC) counts and infection-related biomarkers among the non-severe, severe and critically severe groups (**Table 2**). Sixty-two out of 211 (29.4%) patients had decreased WBC counts below normal levels, and 27 out of 211 (12.8%) patients exhibited increased WBC counts that exceeded normal levels (**Supplementary Figure 5**). Critically severe cases had higher WBC counts than non-severe cases (8.5 *vs*. 4.6×10^9^/L, *p*<0.001; **Figure 9A**). In the non-severe and severe groups, 5 out of 111 (4.5%) patients had increased WBC counts, and 5 of 41 (12.2%) patients exhibited increased WBC counts. In the critically severe patients, 17 of 59 (28.8%) patients had increased WBC counts. A total of 151 out of 211 (71.6%) patients exhibited lower lymphocyte counts than normal levels (**Supplementary Figure 5**). Sixty-five out of 111 (58.6%) non-severe patients had decreased lymphocytes below normal levels, while lymphocyte counts were below normal levels for most severe and critically severe patients (35/41; 51/59). Moreover, the severe and critically severe groups had fewer lymphocytes than the non-severe group (0.8 *vs*. 1.1×10^9^/L, *p*<0.001; 0.7 *vs*. 1.1×10^9^/L, *p*<0.001; **Figure 9B**).

Different subsets of peripheral lymphocytes were further analyzed (**Table 3**). The median absolute count of CD3+ T lymphocytes in non-severe patients (845.2/μL) was within the normal range. The number of CD3+ T lymphocytes was below the lower limit of normal value in both the severe (526.4/μL) and critically severe (370.6/μL) groups. There were significant differences in CD3+ T lymphocyte counts between severe or critically severe patients and non-severe patients (526.4 *vs*. 845.2/μL, *p*<0.01; 370.6 *vs*. 845.2/μL, *p*<0.001; **Figure 9C**). The severe and critically severe cases exhibited lower percentages of CD3^+^ T lymphocytes than the non-severe cases (61.9 *vs*. 67.9%, *p*=0.079; 64.6 *vs*. 67.9%, *p*=0.283; **Figure 9D**). The absolute count of CD4+ T cells in non-severe patients remained in the normal range (503.2/μL). The severe and critically severe groups had significantly decreased numbers of CD4+ T cells compared with the non-severe group (318.6 *vs*. 503.2/μL, *p*<0.05; 213.3 *vs*. 503.2/μL, *p*<0.001; **Figure 9E**). The plasma proportion of CD4+ T cells exceeded the upper limit of the normal value in patients with COVID-19 (38.8%), and showed no significant difference among the three groups (*p*=0.68; **Figure 9F**). The CD8+ T cell count was below the lower limit of normal value in all groups. Severe and critically severe patients had significantly fewer CD8+ T cells than non-severe patients (200.2 *vs*. 311.3/μL, *p*<0.05; 148.3 *vs*. 311.3/μL, *p*<0.001; **Figure 9G**). The plasma proportion of CD8+ T cells in all groups was within the normal range and showed no significant difference among the three groups (*p*=0.688; **Figure 9H**). The CD4+/CD8+ T cell ratio in non-severe and severe patients was within the normal range (1.8; 1.8), and it exceeded the reference range in critically severe patients (2.2). There were no significant differences among the three groups (*p*=0.994; **Table 3**). The median absolute counts of B cells and natural killer (NK) cells in all groups were below the normal levels. The decline in B cells had a more obvious trend in the critically severe group than in the non-severe group (118.5 *vs*. 177.7/μL, *p*<0.01; **Figure 9I**). The mean percentage of B cells was increased in severe and critically severe cases compared with non-severe cases, although there was no significant difference (17.6 *vs*. 15.0%, *p*=0.279; 18.8 *vs*. 15.0%, *p*=0.083; **Figure 9J**). The decline in NK cells was more pronounced in the severe and critically severe groups (139.2 *vs*. 185.6/μL, *p*=0.446; 87.4 *vs*. 185.6/μL, *p*<0.001; **Figure 9K**). The median percentage of NK cells in all groups remained in the normal range. No statistical difference was observed among the three groups (*p*=0.27; **Figure 9L**).

**Table 3:** Comparison of peripheral lymphocyte subpopulations in patients with COVID-19 among non-severe, severe and critically severe groups.

	Normal range	All (n=112)	Non-severe (n=66)	Severe (n=20)	Critically Severe (n=26)	*p* value
**Lymphocyte subsets**						
CD3+ T cells/µL	805-4459	678.1 (324-935)	845.2 (461-1093)	526.4 (251-686)	370.6 (226-510)	<0.001
<910	–	81 (72.3%)	39 (59.0%)	17 (85.0%)	25 (96.2%)	0.01
≥910	–	31 (27.7%)	27 (41.0%)	3 (15.0%)	1 (3.8%)	–
CD3+ T cells, %	38.56-70.06	66.1 (57.0-74.1)	67.9 (61.4-76.6)	61.9 (46.6-74.7)	64.6 (53.9-72.8)	0.235
**T cell subsets**						
CD4+ T cells/µL	345-2350	403.0 (168-557)	503.2 (234-670)	318.6 (126-431)	213.3 (99-291)	<0.001
<536	–	80 (71.4%)	38 (57.6%)	17 (85.0)	25 (96.2%)	0.05
≥536	–	32 (28.6%)	28 (42.4%)	3 (15.0%)	1 (3.8%)	–
CD4+ T cells, %	14.21-36.99	38.8 (28.5-46.8)	39.7 (30.0-46.8)	37.2 (27.9-46.0)	37.8 (26.5-47.6)	0.68
CD8+ T cells/µL	345-2350	253.7 (123-338)	311.3 (164-418)	200.2 (98-309)	148.3 (70-195)	<0.001
<304	–	73 (65.2%)	35 (53.0%)	14 (70.0%)	24 (92.3%)	0.019
≥304	–	39 (34.8%)	31 (47.0%)	6 (30.0%)	2 (7.7%)	–
CD8+ T cells, %	13.24-38.53	25.2 (19.4-30.8)	25.7 (19.8-30.9)	24.1 (18.1-31.9)	25.0 (18.8-30.7)	0.688
CD4+/CD8+ ratio	0.96-2.05	1.9 (1.1-2.3)	1.8 (1.2-2.2)	1.8 (1.0-2.4)	2.2 (1.0-2.4)	0.994
<0.9	–	21 (18.8%)	12 (18.2%)	3 (15.0%)	6 (23.1%)	0.592
0.9-2.0	–	53 (47.3%)	33 (50.0%)	11 (55.0%)	9 (34.6%)	–
≥2.0	–	38 (33.9%)	21 (31.8%)	6 (30.0%)	11 (42.3%)	–
B cells/µL	240-1317	157.5 (64-217)	177.7 (89-233)	141.9 (64-147)	118.5 (31-153)	0.01
<95	–	44 (39.3%)	18 (27.3%)	9 (45.0%)	17 (65.4%)	0.646
≥95	–	68 (60.7%)	48 (72.7%)	11 (55.0%)	9 (34.6%)	–
B cells, %	10.86-28.03	16.3 (8.9-22.5)	15.0 (9.0-20.4)	17.6 (6.3-26.0)	18.8 (9.2-24.4)	0.413
NK cells/µL	210-1514	154.5 (57-206)	185.6 (92-234)	139.2 (64-164)	87.4 (22-120)	<0.001
<107	–	50 (44.6%)	24 (36.4%)	7 (35.0%)	19 (73.1%)	0.013
≥107	–	62 (55.4%)	42 (63.6%)	13 (65.0%)	7 (26.9%)	–
NK cells, %	7.92-33.99	16.1 (7.7-21.3)	15.9 (9.0-20.0)	18.7 (10.4-28.2)	14.8 (4.8-22.2)	0.27

Data are expressed as median (IQR) or n (%). CD4+/CD8+ ratio, the ratio of CD4+ T cells and CD8+ T cells. P values comparing non-severe, severe and critically severe groups are from χ^2^ test, Fisher’s exact test, or Kruskal-Wallis test. P<0.05 was considered as statistically significant.

### Correlation Between Peripheral Lymphocyte Subsets and Disease Severity in COVID-19 Patients

**Figure 10:**
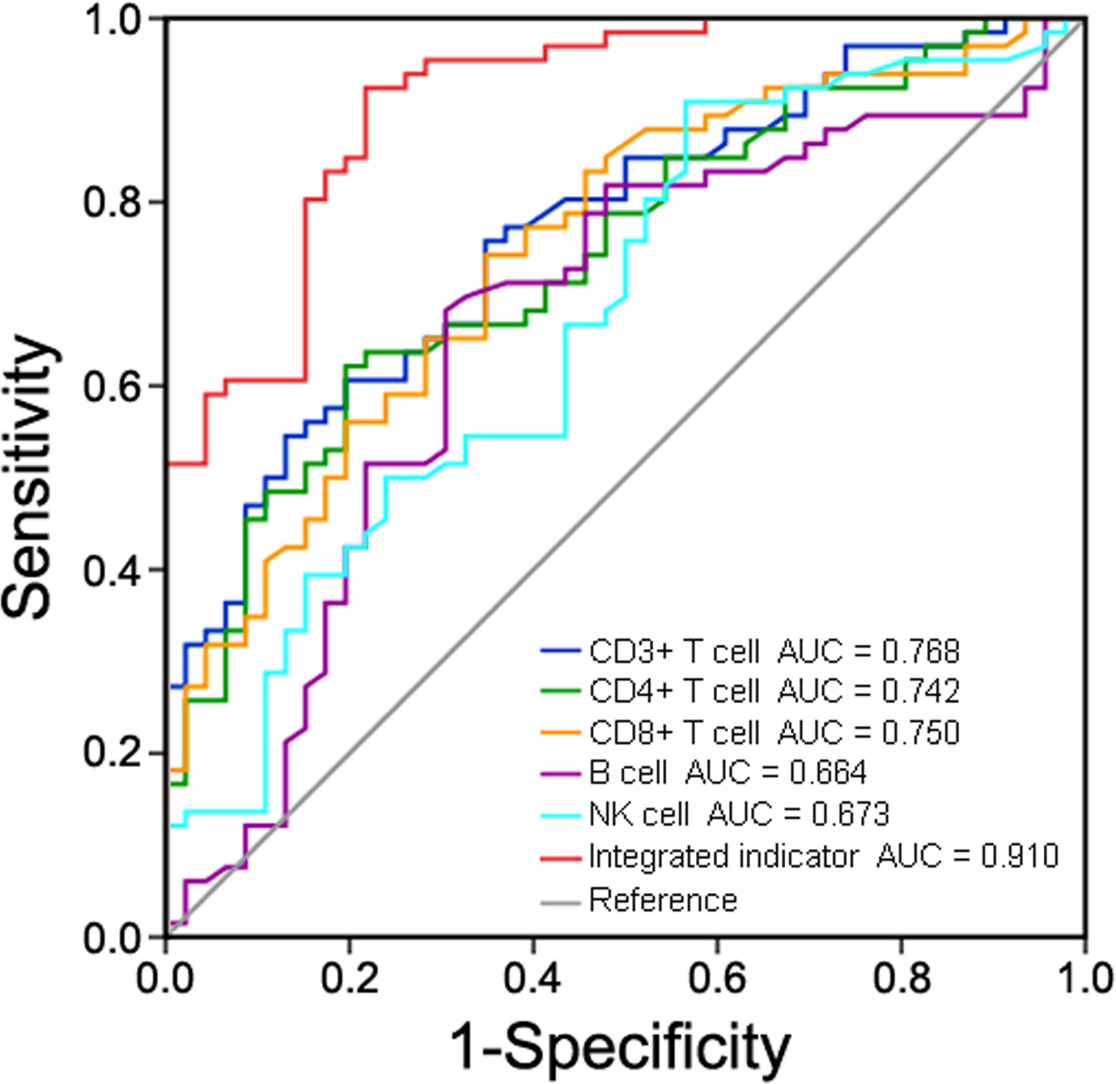
Receiver operating characteristic (ROC) curve analysis of peripheral lymphocyte subsets in predicting disease severity in COVID-19 patients.

ROC curve analysis was performed to assess the correlation between peripheral lymphocyte subsets and disease progression in COVID-19 patients (**Figure 10**). The AUC was 0.768 (95% confidence interval [CI], 0.682-0.854) for CD3+ T cell count, 0.742 (95% CI, 0.651-0.832) for CD4+ T cell count, 0.750 (95% CI, 0.659-0.840) for CD8+ T cell count, 0.664 (95% CI, 0.558-0.770) for B cell count, 0.673 (95% CI, 0.570-0.775) for NK cell count, and 0.910 (95% CI, 0.858-0.963) for the integrated indicator. Bootstrap testing indicated a higher predictive accuracy of the integrated indicator than the CD3+ T cell, CD4+ T cell, CD8+ T cell, B cell or NK cell count individually (*p*<0.01). Taken together, these lymphocyte subpopulations had good accuracy in predicting disease severity in COVID-19 patients.

### Lymphocyte Subset Levels and Inflammatory Status

**Figure 11:**
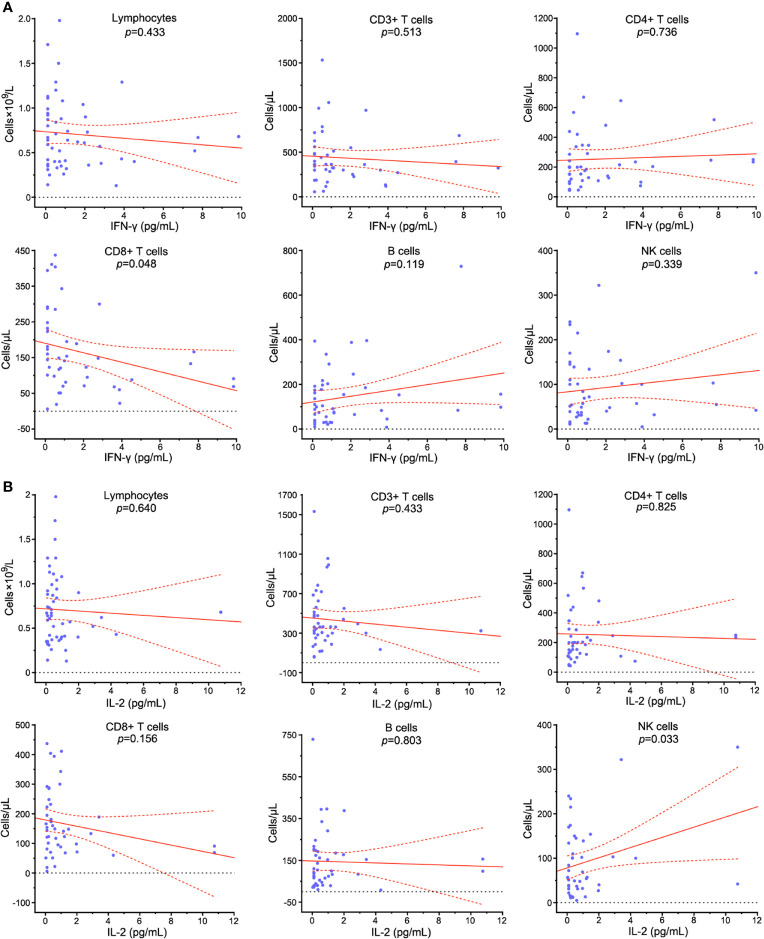
Correlation analysis between peripheral lymphocyte subpopulations and cytokines in COVID-19 patients. The relationships between the counts of lymphocytes, CD3+ T cells, CD4+ T cells, CD8+ T cells, B cells, NK cells and serum levels of IFN-γ **(A)** and IL-2 **(B)** in COVID-19 patients were assessed. Solid line: fitted curve; dashed line: 95% confidence interval (CI) of the fitted curve. A *p* value*<*0.05 was considered as statistically significant.

The inflammatory indicators TNF-α, IL-2, IL-4, IL-6 and IL-10 were abnormal in 12 (23.1%), 5 (9.6%), 16 (30.1%), 99 (86.1%), and 18 (34.6%) patients on admission. The potential correlation between peripheral lymphocyte subpopulations and cytokines was assessed. CD8+ T cell count was negatively correlated with IFN-γ level (*p*=0.048) (**Figure 11A**). NK cell count was positively correlated with IL-2 level (*p*=0.033) (**Figure 11B**). Lymphocytes, CD3+ T cells and B cells showed no significant correlation with IFN-γ (*p*=0.433*, p*=0.513 and *p*=0.119), TNF-α (*p*=0.796, *p*=0.475 and *p*=0.838), IL-2 (*p*=0.640, *p*=0.433 and *p*=0.803), IL-4 (*p*=0.693, *p*=0.509 and *p*=0.716), IL-6 (*p*=0.165, *p*=0.065 and *p*=0.234) and IL-10 (*p*=0.994, *p*=0.422 and *p*=0.773) (**Supplementary Figures 6–9**).

### Independent Predictors and the Prediction Model for Severe and Critically Severe COVID-19

Compared with non-severe patients, patients with older age or higher body temperature before admission were more likely to deteriorate into severe illness (**Table 4**). Male sex was associated with increased odds of both COVID-19 severity levels. Duration of hospitalization was also correlated with increased odds of severe and critically severe cases. Hypertension patients had an approximately 4.0-fold increased risk of disease deterioration. Diabetes and cardiovascular and cerebrovascular diseases were linked with more than 4.7- and 9.5-fold increased risks of severe and critical diseases, respectively. There was a 49.9% increased risk of critical disease for WBC count. A lower lymphocyte count on admission was associated with an increased risk for disease progression. Serum AST levels were associated with increased odds of both COVID-19 severity levels. Serum CREA levels were related to an increased odds of critical diseases in COVID-19 patients. IL-6 was associated with 1.2-fold or greater increased odds of both clinical severity levels.

**Table 4:** Multivariable multinomial logistic regression analysis of risk factors to predict disease severity in COVID-19 patients.

Factor	COVID-19 disease severity^a^			
	Severe, OR (95% CI)	*p* value	Critically severe, OR (95% CI)	*p* value
**Demographics**				
Age, years	1.042 (0.968-1.121)	0.277	1.094 (1.016-1.179)	0.018
Sex (male *vs*. female)	2.393 (1.043-5.494)	0.040	3.572 (1.557-8.197)	0.003
**Timeline after onset of illness, days**				
Duration from illness onset to admission	1.012 (0.921-1.111)	0.807	1.051 (0.961-1.151)	0.275
Duration of hospitalization	1.084 (1.027-1.143)	0.003	1.082 (1.027-1.141)	0.003
**Body temperature, °C**				
Temperature before admission	2.128 (0.934-4.848)	0.072	4.866 (1.835-12.906)	0.001
Temperature at day 15 after admission	1.241 (0.365-4.217)	0.729	2.729 (0.889-8.380)	0.079
**Comorbidity (yes *vs*. no)**				
Hypertension	4.097 (1.604-10.468)	0.003	3.939 (1.612-9.624)	0.003
Diabetes	4.718 (1.237-17.990)	0.023	2.350 (0.597-9.250)	0.222
Cardiovascular and cerebrovascular diseases	2.182 (0.343-13.902)	0.409	9.581 (1.900-48.320)	0.006
Respiratory system diseases	1.674 (0.327-8.574)	0.536	3.184 (0.796-12.732)	0.101
**Complete blood count**				
White blood cell count, ×10^9^/L	1.186 (0.950-1.482)	0.132	1.499 (1.173-1.917)	0.001
Lymphocyte, ×10^9^/L	0.051 (0.004-0.603)	0.018	0.755 (0.336-1.696)	0.496
**Liver and renal function indicators**				
Alanine aminotransferase, U/L	1.004 (0.988-1.019)	0.645	0.993 (0.976-1.012)	0.476
Aspartate aminotransferase, U/L	1.025 (1.001-1.049)	0.040	1.048 (1.024-1.073)	<0.001
Creatinine, μmol/L	1.013 (0.995-1.030)	0.159	1.019 (1.002-1.037)	0.025
**Inflammatory cytokines**				
IFN-γ, pg/mL	1.938 (0.728-5.162)	0.186	1.237 (0.485-3.156)	0.656
TNF-α, pg/mL	0.783 (0.108-5.665)	0.808	0.852 (0.197-3.682)	0.831
IL-2, pg/mL	4.346 (0.684-27.607)	0.119	0.517 (0.095-2.826)	0.447
IL-4, pg/mL	1.916 (0.169-21.766)	0.600	2.887 (0.410-20.330)	0.287
IL-6, pg/mL	1.227 (1.040-1.447)	0.015	1.238 (1.059-1.448)	0.007
IL-10, pg/mL	0.405 (0.169-0.968)	0.042	0.751 (0.513-1.099)	0.140
**Lymphocyte subsets**				
CD3+ T cells/μL	0.979 (0.917-1.045)	0.523	0.959 (0.897-1.025)	0.214
CD3+ T cells, %	0.973 (0.537-1.762)	0.928	1.034 (0.675-1.584)	0.877
B cells/μL	1.001 (0.988-1.014)	0.866	1.012 (0.994-1.031)	0.203
B cells, %	0.983 (0.643-1.503)	0.937	0.884 (0.734-1.066)	0.197
NK cells/μL	0.996 (0.983-1.009)	0.535	1.000 (0.985-1.017)	0.955
NK cells, %	1.058 (0.704-1.589)	0.788	0.977 (0.874-1.092)	0.682
**T cell subsets**				
CD4+ T cells/μL	1.021 (0.957-1.089)	0.524	1.017 (0.951-1.088)	0.621
CD4+ T cells, %	0.995 (0.633-1.564)	0.984	1.034 (0.651-1.642)	0.887
CD8+ T cells/μL	1.025 (0.953-1.103)	0.504	1.061 (0.984-1.144)	0.126
CD8+ T cells, %	1.022 (0.629-1.662)	0.929	0.962 (0.600-1.541)	0.871
CD4+/CD8+ ratio	1.436 (0.301-6.842)	0.650	2.786 (0.570-13.620)	0.206

a Reference level is non-severe. P values were obtained by multivariate logistic analysis. P<0.05 was considered as statistically significant. OR, odds ratio; CI, confidence interval.

## Discussion

In this study, we comprehensively analyzed the clinical characteristics and examined risk factors for disease severity in a patient cohort with COVID-19 in Wuhan, Hubei Province, China. The clinical features of COVID-19 patients enrolled in this study were comparable with those of previous studies. Generally, the patients with COVID-19 in the severe and critically severe groups were of old age and had underlying comorbidities. Baseline diseases such as hypertension, diabetes, cardiovascular and cerebrovascular diseases and respiratory system disorders were associated with an increased risk of disease severity in COVID-19 patients, which was in line with the observations of previous clinical studies (Tian et al., 2020). Male patients tended to have more underlying illnesses than female patients. Therefore, male sex conferred an increased risk of severe illness from COVID-19. The majority of COVID-19 patients presented with fever and chest imaging alterations. Our results demonstrated that higher body temperature before admission might be a risk factor for disease deterioration in COVID-19 patients. Eleven (28.2%) patients still presented with fever even at 15 days after admission. We further revealed that the duration of fever was longer from onset to hospitalization in severe and critically severe cases than in non-severe cases through the analysis of the kinetics of body temperatures, which was consistent with a previous study (Li et al., 2020). All the non-severe patients had normal body temperatures within 10 days after admission. Severe and critically severe patients restored normal body temperature at least 14 days after admission. Accordingly, sustained fever during hospitalization might be an indicator of the severity of the illness in COVID-19 patients.

SARS-CoV-2 could cause dysfunction of the liver and kidney, given the presence of the ACE2 receptor in these tissues (Sungnak et al., 2020; Zou et al., 2020). The pathogenesis of liver and kidney diseases in COVID-19 patients may be attributed to a direct viral cytopathic effect or robust inflammatory response (Blanco-Melo et al., 2020; Huang et al., 2020). Accordingly, elevated levels of serum aminotransferases and CREA have become common clinical signs in COVID-19 patients (Del Zompo et al., 2020; Yang and Yang, 2020). Moreover, abnormalities of liver and renal function were found to be associated with disease deterioration and in-hospital death in COVID-19 patients (Chen et al., 2020; Cheng et al., 2020; Ding et al., 2020). In this study, liver and renal function indicators of patients with COVID-19 were measured at hospital admission. We discovered that severe and critically severe patients had elevated levels of serum ALT, AST and CREA compared to non-severe patients. In critically severe patients, the serum levels of ALT, AST and CREA were higher in non-survivors than in survivors. However, there were no significant differences between survivors and non-survivors (**Supplementary Figure 10**). The multivariate logistic analysis indicated that abnormal liver and renal functions might be risk factors for disease deterioration in patients with COVID-19. The liver and kidney injuries in COVID-19 patients can be attributed to direct cytopathogenic effect of the virus, immune-mediated inflammation, or drug-induced toxicity (Zheng et al., 2020; Ruan et al., 2021). It has been proposed that initially recommended antiviral drugs (e.g., lopinavir/ritonavir) and antibiotics (e.g., moxifloxacin, cephalosporin and carbapenem) were potentially hepatotoxic or nephrotoxic in patients with severe COVID-19 (Soto et al., 2002; Cai et al., 2020a; Cai et al., 2020b; Morales-Alvarez, 2020). The aforementioned medications could increase the risk of liver and kidney injuries in patients with COVID-19. Antiviral drugs and antibiotics are mainly metabolized in the liver, and their metabolites can be found in the urine of patients (Rismanbaf and Zarei, 2020). Liver and kidney damages can affect metabolism, effective concentrations and expected efficacy of these medications. Thus, in the treatment of COVID-19, drug-induced liver and kidney injuries cannot be overlooked. The clinical indicators and complications of patients with COVID-19 must be fully considered in therapy decision making. Moreover, antiviral and antibiotic treatments should be used with great caution. In addition to actively treating COVID-19, it is also imperative to carefully monitor the occurrence and progression of liver and kidney injuries. The usage of hepatoprotective or nephroprotective drugs are recommended for patients with severe liver or kidney injury (Zhang et al., 2020a).

Lymphocytes and their subsets play a critical role in the preservation of immune system function as well as viral clearance (Yap et al., 1978; Sitati and Diamond, 2006). Recent reports showed that lymphocyte counts were reduced in most severe patients with COVID-19 but remained within the normal range in non-severe patients (Chang et al., 2020; Wang et al., 2020). We found that lymphopenia was common in patients with COVID-19 (151, 71.6%), and lymphopenia was more pronounced in severe and critically severe patients than in non-severe patients (0.8 *vs*. 1.1×10^9^/L, *p*<0.001; 0.7 *vs*. 1.1×10^9^/L, *p*<0.001). SARS-CoV-2 infection can cause impairment of the immune system during disease progression (Qin et al., 2020). SARS-CoV-2 is able to replicate more efficiently in patients with compromised immune systems, thereby resulting in severe disease in these patients (Corse et al., 2020). We found that there were negative associations between the age of patients and the numbers of lymphocyte subsets, especially lymphocytes, CD3+ T cells, CD4+ T cells, CD8+ T cells and B cells (**Supplementary Figure 11**). These results demonstrated that elderly patients were prone to develop severe disease. Therefore, it is of great importance to closely track disease progression and provide timely treatment for elderly patients.

The counts of lymphocyte subpopulations (CD3+ T cells, CD4+ T cells, CD8+ T cells, B cells and NK cells) were reported to decline in the peripheral blood of patients with COVID-19 (Liu et al., 2020a; Yu and Yang, 2020). In the present study, the profile of lymphocyte subpopulations in the peripheral blood of COVID-19 patients was also characterized. We revealed that most of the COVID-19 patients had lower than normal levels of CD3+ T cells, CD4+ T cells, CD8+ T cells, B cells and NK cells. Thus, our results were in accordance with previous reports. Notably, the decline in CD3+ T cells, CD4+ T cells, CD8+ T cells, B cells and NK cells was more marked in critically severe patients with COVID-19 than in non-severe patients. Thus, SARS-CoV-2 represses cellular immunity in COVID-19 patients.

Cytokine storms play a critical role in the acute lung injury of severe patients with COVID-19 (Zhang et al., 2020b). It was reported that the levels of inflammatory markers were remarkably higher in severe patients than in non-severe patients, suggesting that the cytokine storm was positively correlated with disease severity (Han et al., 2020). Contrary to previous findings (Diao et al., 2020; Huang et al., 2020; Liu et al., 2021), severe and critically severe patients displayed lower levels of TNF-α, IL-2, IL-4 and IL-10 than non-severe patients in this study. Moreover, the serum levels of IFN-γ, TNF-α, IL-2, IL-4 and IL-10 were not correlated with disease severity among COVID-19 patients. It remains to be defined why COVID-19 patients had severe and critical clinical characteristics independent of circulating levels of inflammatory cytokines (IFN-γ, TNF-α, IL-2, IL-4 and IL-10). A small quantity (52/211, 24.6%) of patients was included in the examination of these cytokines. Links between the dynamics of cytokines and disease progression remain to be elucidated. Further study on the cytokine profile of COVID-19 patients would be helpful to comprehend COVID-19 pathophysiology and open up new avenues towards cytokine-targeted therapies offered to COVID-19 patients.

High IL-6 levels in COVID-19 patients are considered an immunological sign of ongoing cytokine storms (Ruan et al., 2020). IL-6 functions as a predominant proinflammatory mediator for the induction of acute phase responses, resulting in a series of systemic changes, including fever and hemodynamic effects (Quartuccio et al., 2021). The activation of IL-6 signaling also induces the expression of vascular endothelial growth factor (VEGF) in endothelial cells, enhances vascular permeability and inhibits myocardial contractility (Mehta et al., 2020). These events finally cause organ damage and death in COVID-19 patients. Existing evidence has suggested a correlation between high IL-6 levels and the progression of COVID-19 severity (Gong et al., 2020; Liu et al., 2020). It was proposed that IL-6 might be a potential biomarker in predicting clinical outcome in critically ill COVID-19 patients (Gorham et al., 2020). In addition, the high IL-6 levels in COVID-19 patients were associated with a decrease in CD4+ and CD8+ T cells (Xu et al., 2020). Our results were consistent with those of previous studies. We found that the median concentration of IL-6 in COVID-19 patients (72.0 [IQR, 8.5-80.9] pg/mL) exceeded the reference value. IL-6 levels were significantly higher in severe and critically severe patients than in non-severe patients (33.3 *vs*. 21.8 pg/mL, *p*<0.05; 140.6 *vs*. 21.8 pg/mL, *p*<0.001). Notably, the concentration of IL-6 was significantly higher in non-survivors than in survivors (*p*<0.01; **Supplementary Figure 12**), suggesting that IL-6 has predictive value for mortality in COVID-19 patients. Based on these results, we postulated that COVID-19 patients with increased IL-6 levels should be expeditiously identified and receive effective intervention.

However, several limitations of this study should be noted. First, the levels of serum biochemical indexes, cytokines, and lymphocyte subpopulations were not tested at standard intervals. Second, because of the retrospective analysis, the information of some COVID-19 cases was incomplete. Third, all hospitalized COVID-19 patients were transferred to Leishenshan Hospital for further medical management, according to the unified arrangement of the Wuhan municipal government. Clinical data of COVID-19 patients were not collected after transfer to Leishenshan Hospital. These limitations may affect the accuracy of these clinical and laboratory parameters to predict disease progression and clinical outcome in COVID-19 patients. Consecutive surveillance of clinical and immunopathologic characteristics in larger cohorts of COVID-19 patients would be necessary to validate our conclusion. An in-depth investigation of cytokine levels and dysfunction of the immune system during COVID-19 progression would help to identify COVID-19 patients who may benefit from cytokine blocker-based therapy.

In summary, this study provided a systematic characterization of demographic data and clinical and immunological characteristics among COVID-19 patients. The results indicated that advanced age, male sex and baseline comorbidities might increase the risk of developing severe illness in COVID-19 patients. Moreover, body temperature before admission, sustained fever status, the length of hospital stay, higher WBC counts, elevated levels of AST, CREA and IL-6, and lymphopenia on admission tended to be independent factors for predicting disease severity in COVID-19 patients. This study emphasized the importance of follow-up surveillance of the clinical and immunopathologic indicators in patients with COVID-19 during hospitalization and may aid clinicians in providing prompt management of COVID-19 patients.

## Data Availability Statement

The original contributions presented in the study are included in the article/**Supplementary Material**. Further inquiries can be directed to the corresponding authors.

## Ethics Statement

The studies involving human participants were reviewed and approved by the Medical Ethics Committee of Zhongnan Hospital of Wuhan University (NO. 2020026). All data were collected anonymously and recorded following the international standards for the protection of privacy and personal information. Written informed consent was waived by the institutional review board for emerging infectious disease.

## Author Contributions

MW and YF analyzed and interpreted the data. YC and WC contributed to clinical data collection and literature searches. MW drew the figures and wrote the manuscript. KW, JC and XH supervised the project, provided crucial ideas and revised the manuscript. All authors contributed to the article and approved the submitted version.

## Funding

This work was supported by the National Natural Science Foundation of China (No. 81701991) and the Applied Basic Research Program of Qingdao, China (No. 17-1-1-59-jch).

## Conflict of Interest

The authors declare that the research was conducted in the absence of any commercial or financial relationships that could be construed as a potential conflict of interest.
