# Supplementary material for: Association of Clinical and Immunological Characteristics With Disease Severity and Outcomes in 211 Patients With COVID-19 in Wuhan, China
Source: Front Cell Infect Microbiol. 2021 May 28;11:667487. doi: 10.3389/fcimb.2021.667487 (PMC8195246; doi:10.3389/fcimb.2021.667487)
Supplement: Supplementary file 1 [file DataSheet_1.pdf]

Supplementary Material

1 Supplementary Tables

Supplementary Table 1. Treatments of patients with COVID-19 during hospitalization.

Treatments	All (n=211)	Non-severe (n=111)	Severe (n=41)	Critically severe (n=59)	*p* value
**Antivirals**	209 (99.1%)	111 (100.0%)	41 (100.0%)	57 (96.6%)	0.074
Arbidol	68 (32.5%)	38 (34.2%)	13 (31.7%)	17 (29.8%)	0.769
Oseltamivir	143 (68.4%)	71 (64.0%)	34 (82.9%)	38 (66.7%)	0.069
Lopinavir/ritonavir	57 (27.3%)	27 (24.3%)	12 (29.3%)	18 (31.6%)	0.645
**Glucocorticoids**					
Methylprednisolone sodium succinate	69 (32.7%)	31 (27.9%)	16 (39.0%)	22 (37.3%)	0.293
**Antibiotics**	105 (49.8%)	54 (48.6%)	24 (58.5%)	27 (45.8%)	0.428
Moxifloxacin	93 (88.6%)	52 (96.3%)	20 (83.3%)	21 (77.8%)	0.296
Cephalosporin	49 (46.7%)	16 (29.6%)	14 (58.3%)	19 (70.4%)	0.006
Carbapenem	37 (35.2%)	15 (27.8%)	10 (41.7%)	12 (44.4%)	0.235
**Traditional Chinese medicine**					
Lianhuaqingwen capsules	9 (4.3%)	4 (3.6%)	3 (7.3%)	2 (3.4%)	0.559
**Intravenous immunoglobulin therapy**	66 (31.3%)	29 (26.1%)	15 (36.6%)	22 (37.3%)	0.235
**Continuous renal replacement therapy (CRRT)**	5 (2.4%)	1 (0.9%)	1 (2.4%)	3 (5.1%)	0.233
**Oxygen inhalation**	141 (66.8%)	66 (59.5%)	32 (78.0%)	43 (72.9%)	0.049
**Non-invasive mechanical ventilation**	53 (25.1%)	8 (7.2%)	24 (58.5%)	21 (35.6%)	<0.001
**Invasive mechanical ventilation**	6 (2.8%)	0 (0.0%)	2 (4.9%)	4 (6.8%)	0.028
**Extracorporeal membrane oxygenation (ECMO)**	2 (0.9%)	0 (0.0%)	0 (0.0%)	2 (3.4%)	0.074

Data are expressed as n (%). P values comparing non-severe, severe and critically severe groups are from χ^2 test, Fisher's exact test, or Kruskal-Wallis test. P<0.05 was considered as statistically significant.

Supplementary Table 2. Comparison of comorbidity incidence between male and female patients with COVID-19.

	All (n=211)	Male (n=101)	Female (n=110)	*p* value
**Comorbidity**	74 (35.1%)	43 (42.6%)	31 (28.2%)	<0.001
Hypertension	53 (71.6%)	30 (69.8%)	23 (74.2%)	0.006
Diabetes	28 (37.8%)	16 (37.2%)	12 (38.7%)	0.043
Cardiovascular and cerebrovascular diseases	25 (33.8%)	14 (32.6%)	11 (35.5%)	0.083
Respiratory system diseases	15 (20.3%)	11 (25.6%)	4 (12.9%)	0.006

Data are expressed as n (%). P values indicate differences between male and female patients. P<0.05 was considered as statistically significant.

2 Supplementary Figures

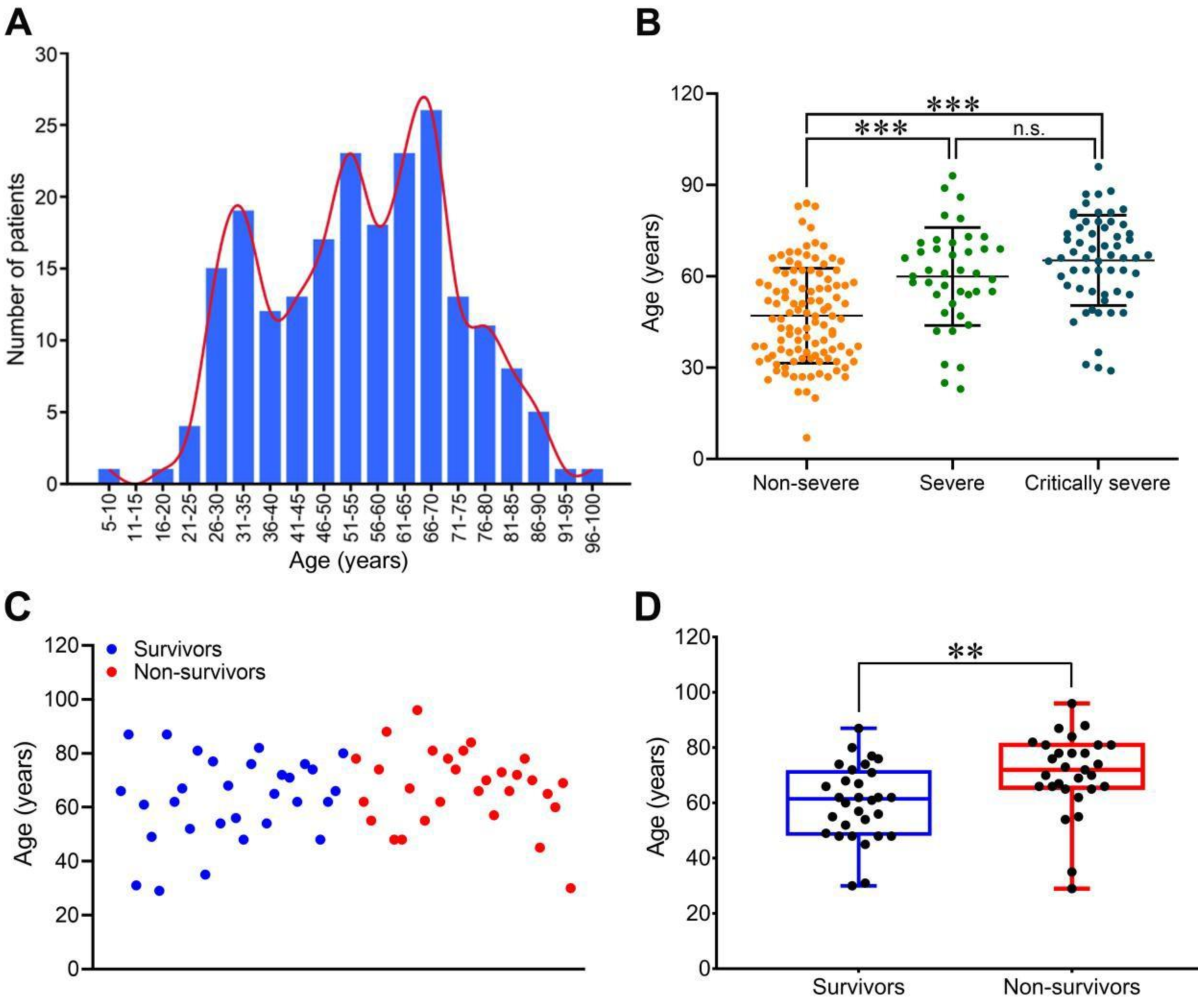

Supplementary Figure 1. Comparison of age characteristics among non-severe, severe and critically severe patients with COVID-19. **(A)** Age distribution of 211 COVID-19 patients enrolled in this study. **(B)** Comparison of the median ages among non-severe, severe and critically severe patients. **(C)** Age distribution of survivors and non-survivors in the critically severe group. **(D)** Comparison of the median ages between survivors and non-survivors in the critically severe group. ***p*<0.01; ****p*<0.001; n.s., not significant.

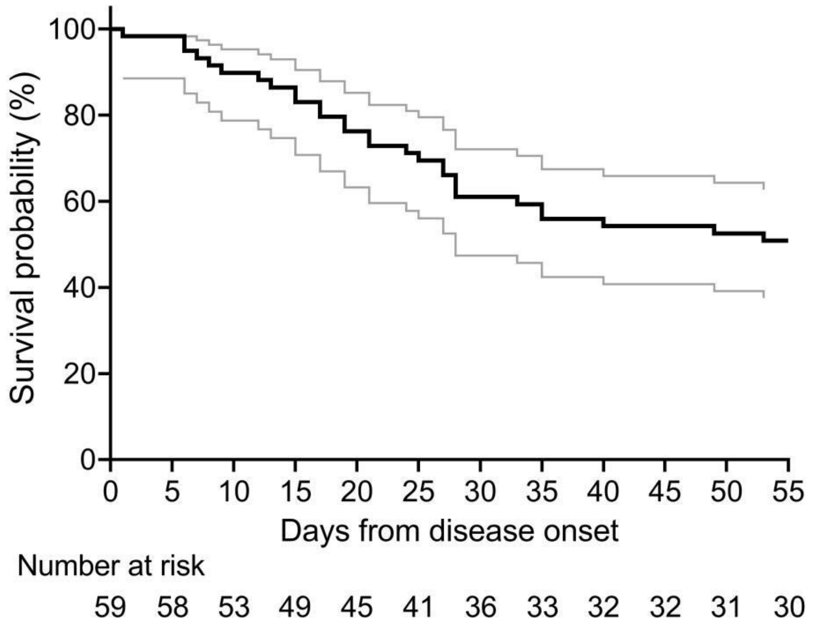

Supplementary Figure 2. Survival curves of 59 critically severe patients with COVID-19. Gray lines represent 95% confidence intervals (CIs).

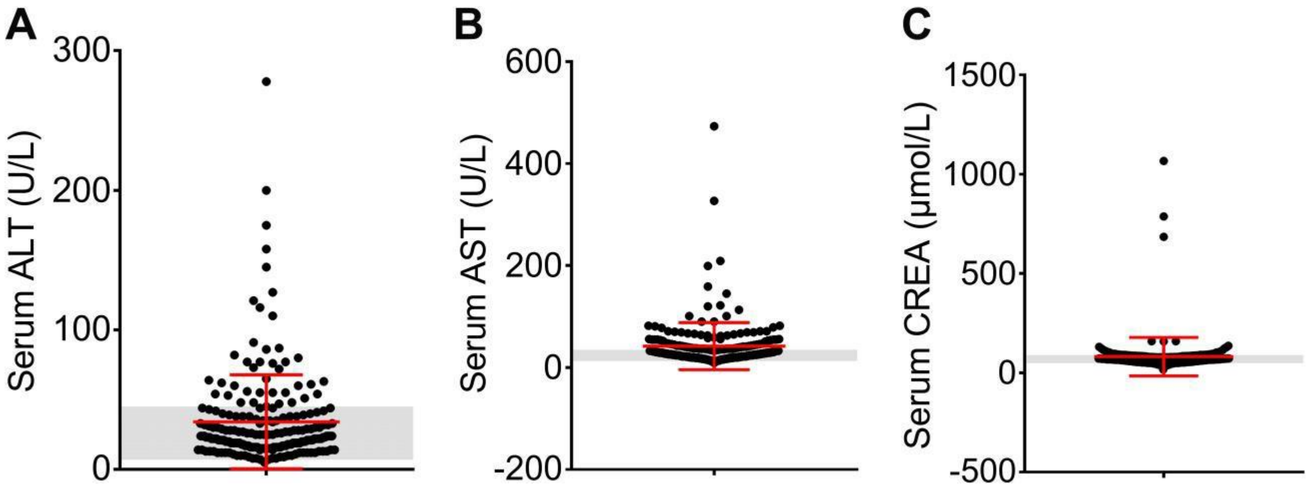

Supplementary Figure 3. The profile of blood biochemical parameters in COVID-19 patients. The concentrations of ALT **(A)**, AST **(B)** and CREA **(C)** in the peripheral blood of COVID-19 patients were assessed. ALT, alanine aminotransferase; AST, aspartate aminotransferase; CREA, creatinine. The normal range for each parameter is shaded in grey.

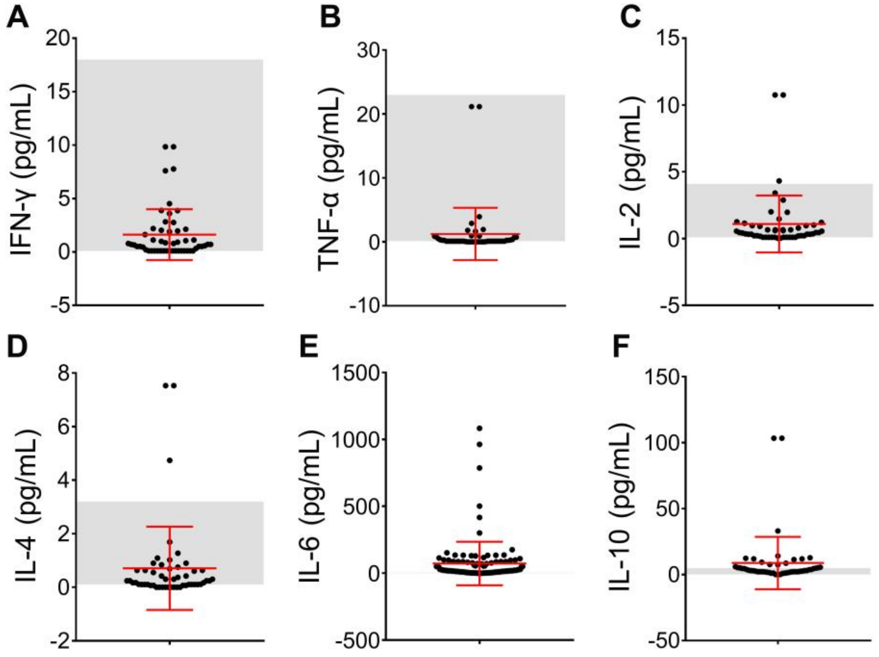

Supplementary Figure 4. The serum levels of various cytokines in COVID-19 patients. The concentrations of IFN-γ **(A)**, TNF-α **(B)**, IL-2 **(C)**, IL-4 **(D)**, IL-6 **(E)** and IL-10 **(F)** in the serum of COVID-19 patients were evaluated. IFN-γ, interferon-γ; TNF-α, tumor necrosis factor-α; IL-2, interleukin-2; IL-4, interleukin-4; IL-6, interleukin-6; IL-10, interleukin-10. The reference ranges are highlighted in grey.

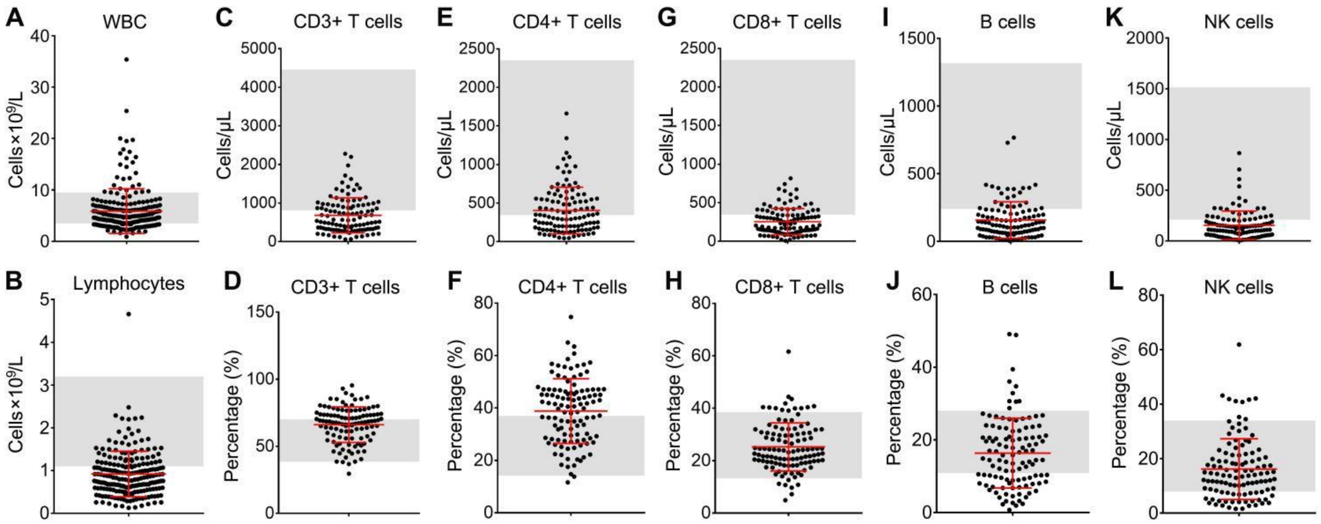

Supplementary Figure 5. Analysis of peripheral lymphocyte subsets in COVID-19 patients. Absolute cell counts of WBC **(A)**, lymphocytes **(B)**, CD3+ T cells **(C)**, CD4+ T cells **(E)**, CD8+ T cells **(G)**, B cells **(I)** and NK cells **(K)** in COVID-19 patients were analyzed by flow cytometry. The percentages of CD3+ T cells **(D)**, CD4+ T cells **(F)**, CD8+ T cells **(H)**, B cells **(J)** and NK cells **(L)** were also detected. WBC, white blood cell; NK, natural killer. The normal ranges are shaded in grey.

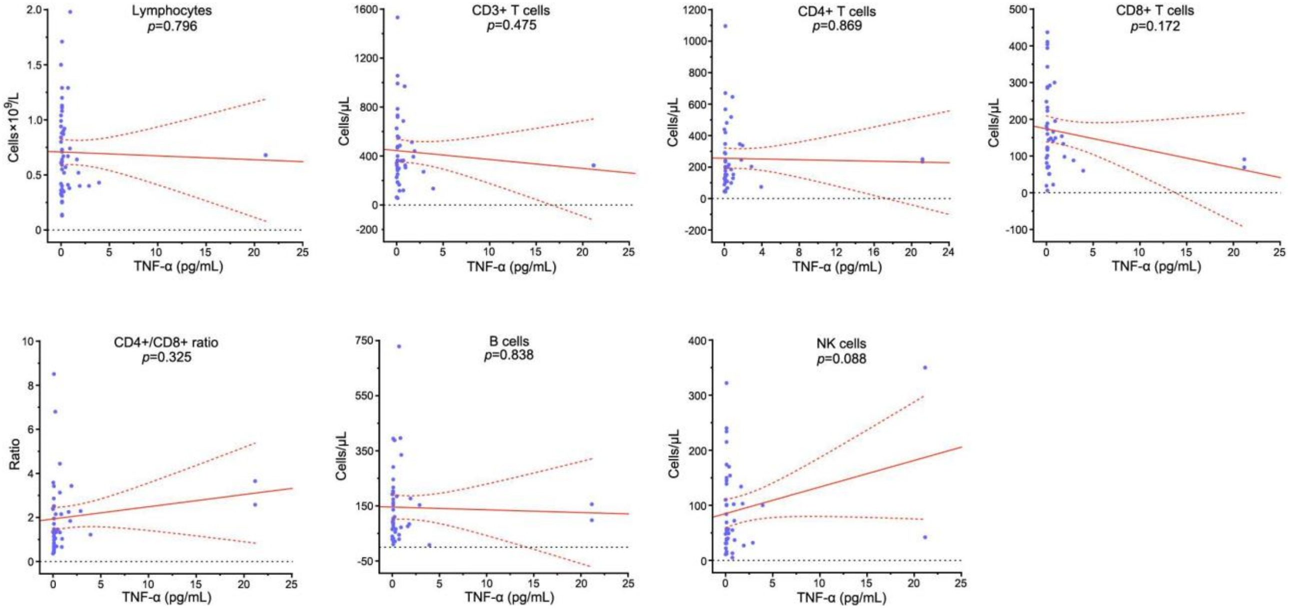

Supplementary Figure 6. Correlation analysis between peripheral lymphocyte subpopulations and TNF-α in COVID-19 patients. Solid line: fitted curve; dashed line: 95% confidence interval (CI) of the fitted curve.

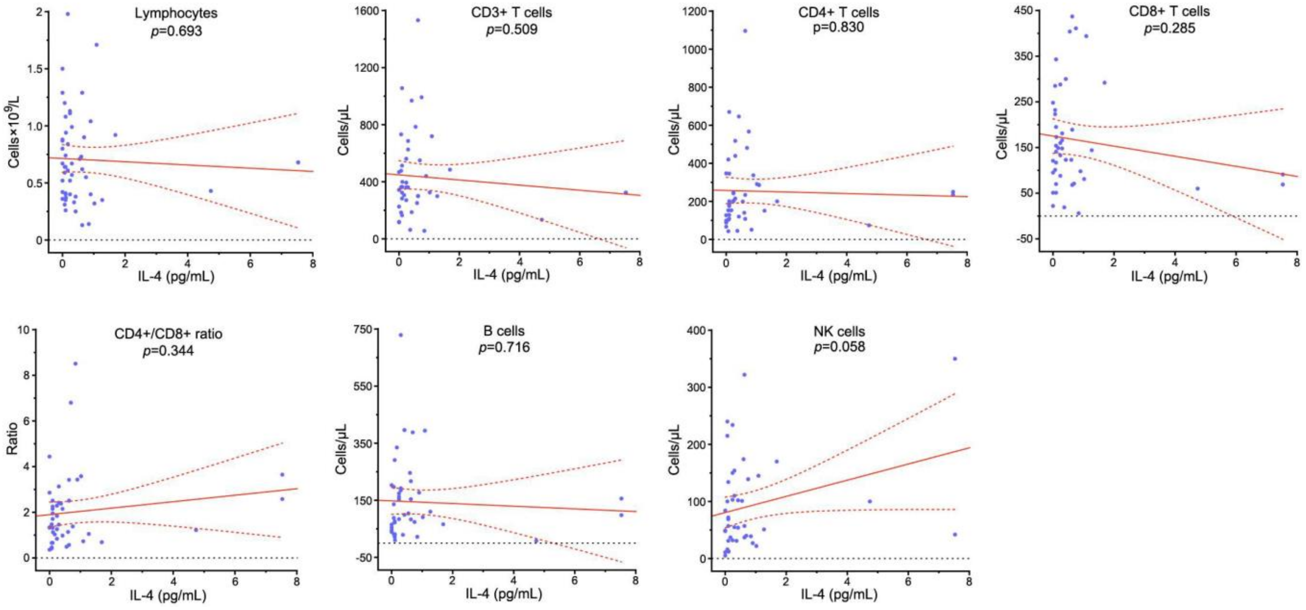

Supplementary Figure 7. Correlation analysis between peripheral lymphocyte subpopulations and IL-4 in COVID-19 patients. Solid line: fitted curve; dashed line: 95% confidence interval (CI) of the fitted curve.

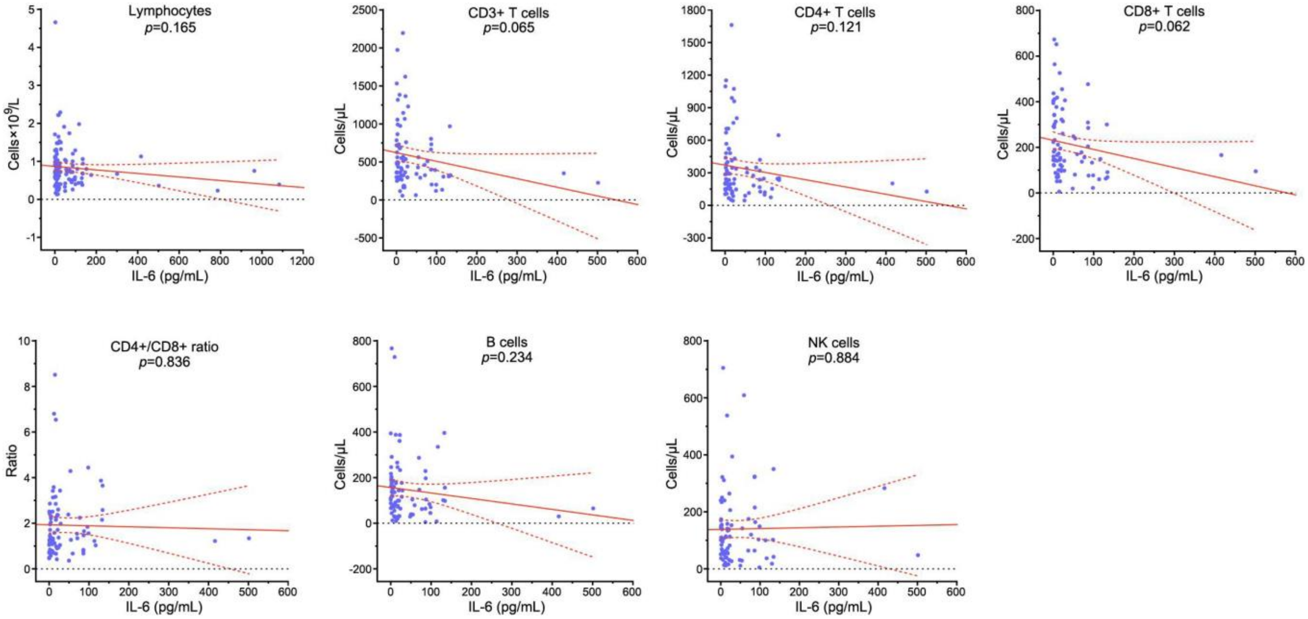

Supplementary Figure 8. Correlation analysis between peripheral lymphocyte subpopulations and IL-6 in COVID-19 patients. Solid line: fitted curve; dashed line: 95% confidence interval (CI) of the fitted curve.

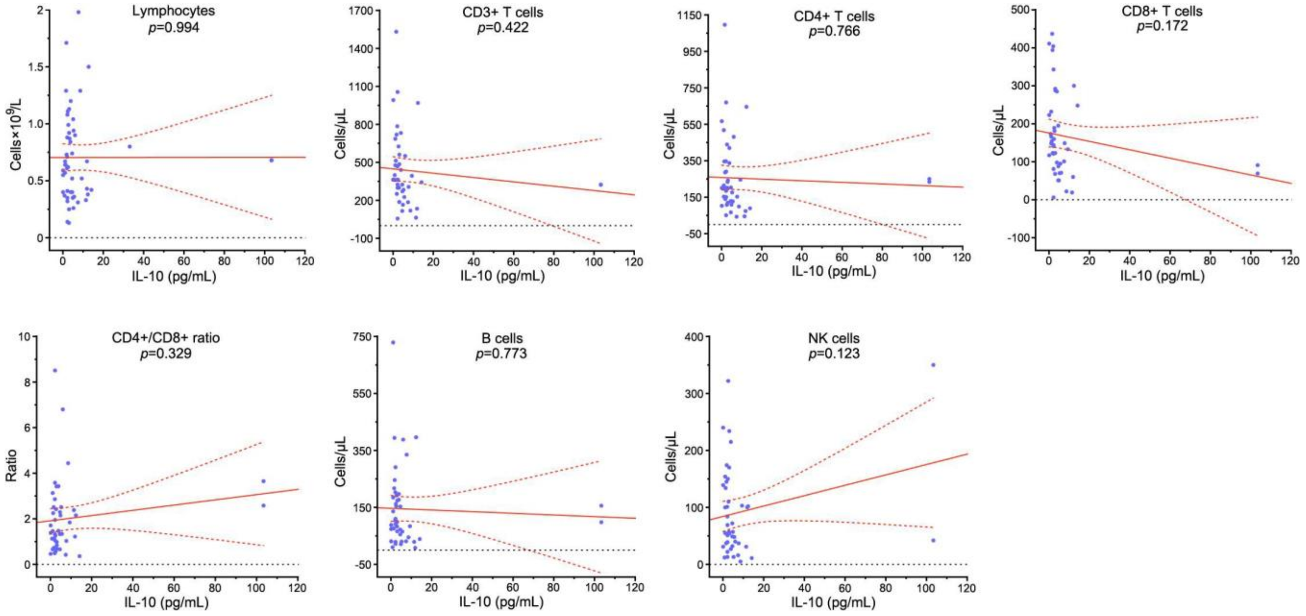

Supplementary Figure 9. Correlation analysis between peripheral lymphocyte subpopulations and IL-10 in COVID-19 patients. Solid line: fitted curve; dashed line: 95% confidence interval (CI) of the fitted curve.

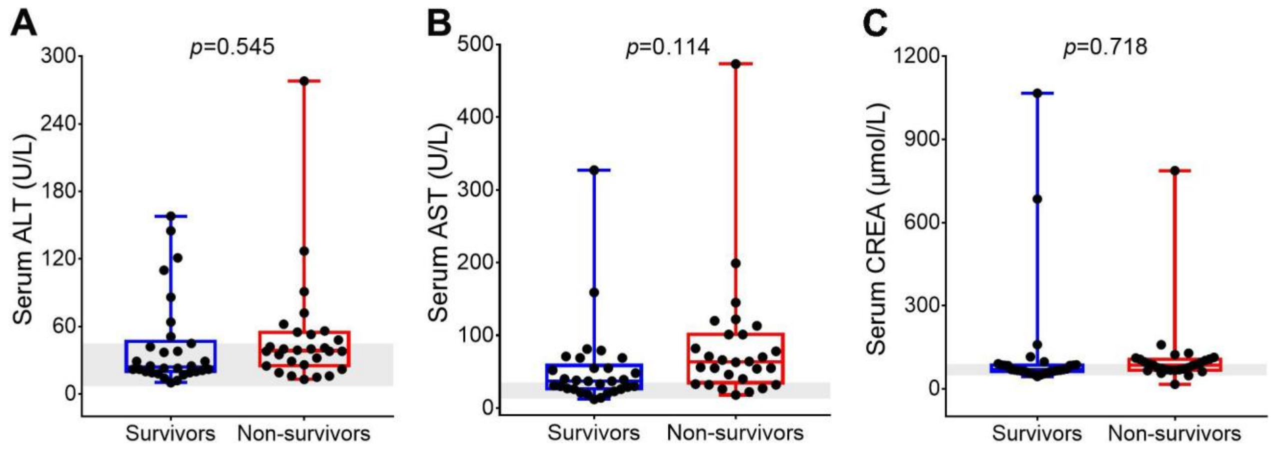

Supplementary Figure 10. Comparison of blood biochemical parameters between survivors and non-survivors in the critically severe group. The levels of ALT **(A)**, AST **(B)** and CREA **(C)** were compared between survivors and non-survivors. ALT, alanine aminotransferase; AST, aspartate aminotransferase; CREA, creatinine. The normal ranges are shaded in grey.

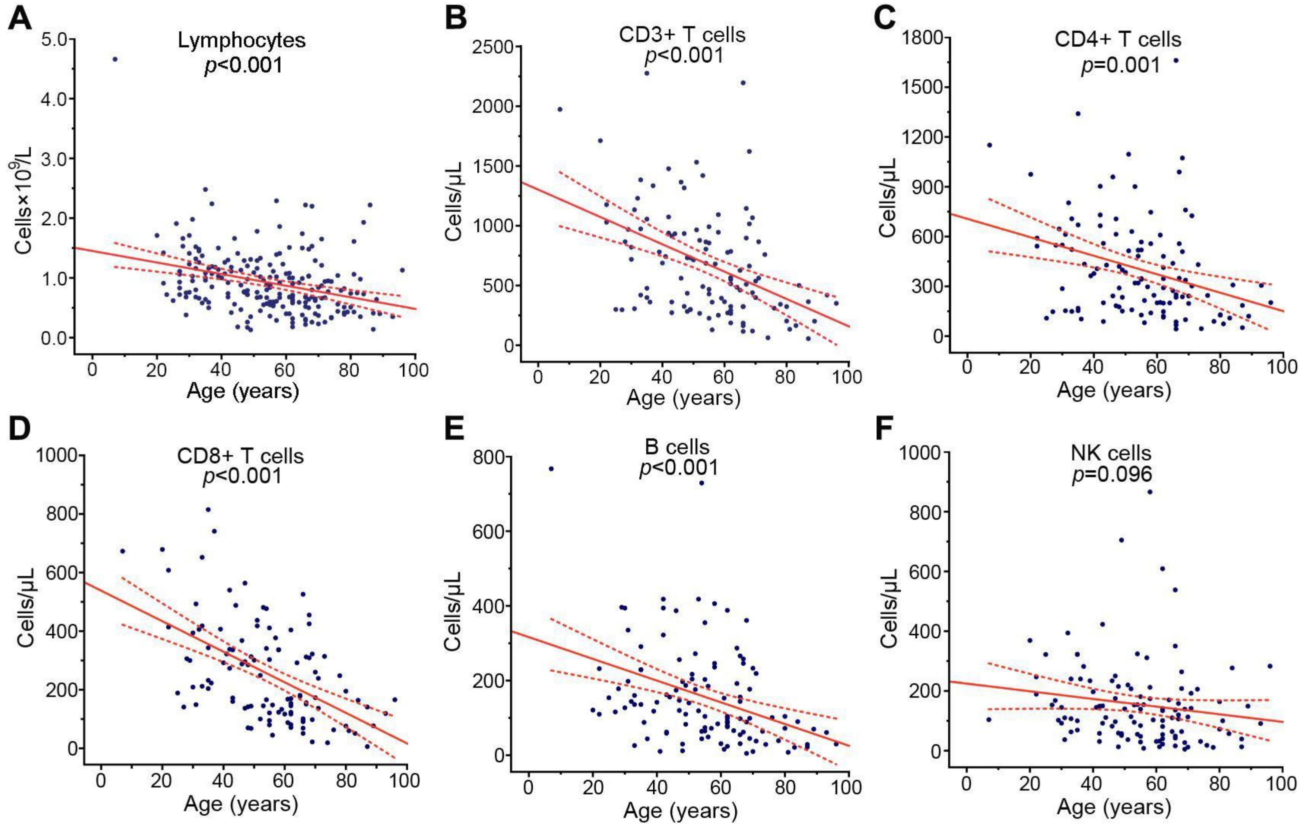

Supplementary Figure 11. Correlation analysis between the age of patients and lymphocyte subpopulation counts. Correlations were investigated between the age of patients and the absolute numbers of **(A)** lymphocytes (p<0.001), **(B)** CD3+ T cells (p<0.001), **(C)** CD4+ T cells (p=0.001), **(D)** CD8+ T cells (p<0.001), **(E)** B cells (p<0.001), and **(F)** NK cells (p=0.096). Solid line: fitted curve; dashed line: 95% confidence interval (CI) of the fitted curve.

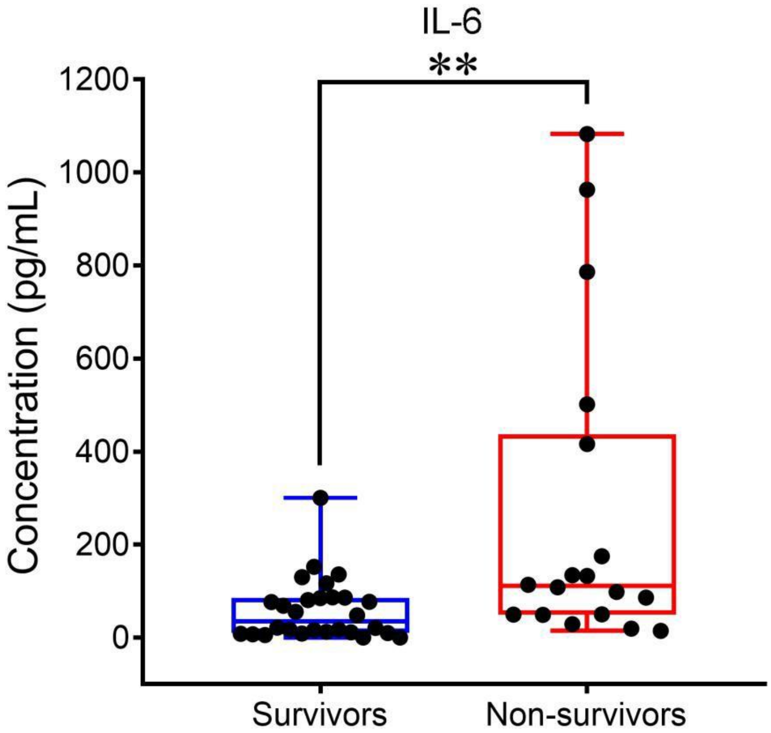

Supplementary Figure 12. Comparison of the IL-6 level in the peripheral blood of survivors and non-survivors in the critically severe group. $^{**}p<0.01$.
